# Supplementary material for: PlasGO: enhancing GO-based function prediction for plasmid-encoded proteins based on genetic structure
Source: Gigascience. 2024 Dec 20;13:giae104. doi: 10.1093/gigascience/giae104 (PMC11659980; doi:10.1093/gigascience/giae104)
Supplement: giae104_Supplemental_File [file giae104_supplemental_file.pdf]

Supplementary information for “PlasGO: enhancing GO-based function prediction for plasmid-encoded proteins based on genetic structure"

Yongxin Ji, Jiayu Shang, Jiaojiao Guan, Wei Zou, Herui Liao, Xubo Tang, Yanni Sun

Electrical Engineering Department, City University of Hong Kong, Kowloon, Hong Kong SAR

October 2024

1 Supplementary Methods: calculation of RR Loss and selection of nominal-format GO term predictions

To compute $\mathcal{L}_{RR}$, the confidence scores output in a batch are initially sorted in descending order. They are then divided into two groups, with a ratio of 70% for the high-confidence group and 30% for the low-confidence group. $\mathcal{L}_{RR}$ is designed to optimize the difference between the mean of the high-confidence group (μ_h) and the mean of the low-confidence group (μ_l), with the goal of approaching a specified hyperparameter δ_1 ($\delta_1 = 0.15$ by default):

$$\mathcal{L}_{RR} = max\left\{0, \delta_1 - (\mu_h - \mu_l)\right\} \tag{1}$$

Importantly, alongside the predicted probabilities P, the classifier module also outputs the confidence scores C to assist in determining the GO term predictions in the format of nominal data, such as $protein_A : [GO_i, GO_j, GO_k]$. To illustrate, we demonstrate the determination of $protein_A$'s annotation with GO term i using the predicted probability P_{Ai} and confidence score C_{Ai}. Since positive proteins are infrequent for most GO terms, the trained model tends to be conservative in predicting positives. Consequently, if P_{Ai} exceeds a predefined cutoff δ_2 ($\delta_2 = 0.425$ by default), the prediction is considered confident enough, irrespective of the value of C_{Ai}, and GO term i is assigned directly to $protein_A$. Alternatively, GO term i is assigned to $protein_A$ only when both conditions, namely $P_{Ai} > \delta_3$ and $C_{Ai} > \delta_4$, are satisfied ($\delta_3 = 0.3$ and $\delta_4 = 0.95$ by default).

2 Exploring the protein length limit of 1Kbp for training PlasGO

We restricted the protein length to 1Kbp for training PlasGO because this limit is computationally efficient for the Transformer architecture, a common practice followed by many state-of-the-art protein-related methods such as ESM [6] and PFresGO [7]. Another reason is that the average length of all RefSeq plasmid-encoded proteins is 293bp, with only 2.18% of them larger than 1Kbp, indicating that removing these larger proteins would not result in a significant loss of features for PlasGO to learn. Nonetheless, **in the prediction phase or when utilizing our PlasGO tool, no length restrictions are imposed.** Therefore, PlasGO can accept users' input proteins exceeding 1K in length and predict their functions drawing from the knowledge acquired from shorter proteins. To evaluate this capability, we conducted an experiment where we predicted GO terms for the larger proteins without further training or fine-tuning. The performance comparison between PlasGO and the other top three tools is presented in Supplementary Table S1.

Supplementary Table S1. Performance comparison between PlasGO and the other top three tools on proteins larger than 1Kbp.

Method	GO category	F_{max}	AUPR
PlasGO	MF	0.8407	0.5652
	BP	0.8741	0.6764
	CC	0.9305	0.8321
PFresGO	MF	0.8091	0.5603
	BP	0.8539	0.5916
	CC	0.8995	0.7999
DeepGOPlus	MF	0.6885	0.2993
	BP	0.7058	0.3756
	CC	0.8417	0.4972
TM-Vec	MF	0.8051	0.522
	BP	0.8718	0.5381
	CC	0.9275	0.5485

PlasGO exhibits the best overall performance on proteins larger than 1Kbp. This indicates the potential for our method to generalize to plasmid-encoded proteins of varying lengths.

3 Methods employed to prevent overfitting for the PlasGO model

1. **Dropout:** dropout layers were applied after the token embedding layers, the multi-head self-attention layers, the feed-forward network within the Transformer encoders, and the contextualized embeddings learned by the BERT module;

2. **Model simplification:** While BERT was utilized to capture plasmid modular patterns, we opted for its hyperparameters aligned with the size of the GO term dataset. For instance, we employed 4 Transformer encoder layers for the Molecular Function (MF) and Biological Process (BP) categories and 2 layers for the Cellular Component (CC) category. This decision was based on the relatively smaller dataset and label size for the CC category;

3. **Regularization technique:** a rank regularization (RR) loss is integrated into the total loss function. This regularization method, elaborated in Supplementary Section S1, aimed to enhance the model's capacity to differentiate between low-confidence and high-confidence predictions. Additionally, the model was prevented from overfitting by discouraging the assignment of high confidence scores to all predictions;

4. **Early stopping:** the training process will stop if the performance on the validation begins to deteriorate;

5. **Cross-validation:** a 5-fold cross-validation benchmark experiment was carried out, as detailed in Supplementary Section S9.

4 Evaluation of computational costs and resource requirements for PlasGO

The PlasGO model is structured as a flexible architecture, featuring a core BERT module integrated with raw per-protein embeddings from various protein language models (PLMs), including models from the ProtTrans family and the ESM family. As demonstrated in the "Ablation studies" experiment section, PlasGO achieves robust performance across a spectrum of PLMs with varying parameter counts. Hence, we tested the maximum GPU memory usage and the runtime for both the training and prediction phases of PlasGO built using the standard ProtT5 model and three alternatives, more lightweight ESM models.

Specifically, each group was trained with a batch size of 32 for 10 epochs. For the prediction phase, we randomly chose 1,000 test plasmids encoding 72,634 proteins. Both the training and prediction involved two steps: the initial preprocessing step to extract per-protein embeddings with PLM, followed by the second step to run the PlasGO model. Notably, all experiments were conducted on a single NVIDIA GeForce RTX 3090 Blower 24G graphics card.

Supplementary Table S2. Comprehensive breakdown of computational resources (maximum GPU memory usage and runtime) for each phase of PlasGO tested using a single NVIDIA RTX 3090 GPU.

PLM	Training phase		Prediction phase	
(# of parameters)	Preprocessing	Run PlasGO	Preprocessing	Run PlasGO
ProtT5 (3B)	23hr (7.36GB)	65min (0.85GB)	1hr15 min (7.36GB)	5.6sec (0.54GB)
ESM-2 (150M)	1hr7min (0.82GB)	64.6min (0.84GB)	4min (0.82GB)	5.5sec (0.52GB)
ESM-2 (35M)	33min (0.72GB)	63min (0.84GB)	110sec (0.72GB)	5.4sec (0.5GB)
ESM-2 (8M)	23min (0.63GB)	62.4min (0.83GB)	75sec (0.63GB)	5.4sec (0.5GB)

As shown in Supplementary Table S2, we can conclude that regardless of the employed PLMs, PlasGO requires minimal computational resources, particularly during the prediction phase. This efficiency stems from PlasGO's core module, structured as a lightweight BERT model with a fixed hidden size of 512 and a small number of Transformer layers (2 for the Cellular Component category and 4 for the other two). Conversely, the preprocessing step could require increased computational resources when employing a large PLM. Since we have provided all training codes, users can readily train their custom models using PLMs that align with their computational capabilities. For instance, users with limited GPU memory could opt for an ESM model with 8 or 35 million parameters to integrate with PlasGO. Additionally, leveraging a knowledge distillation-based protein embedding method, such as MTDP [9], offers a good alternative that significantly reduces resource demands while maintaining comparable performance. Finally, for users without a GPU, they can still annotate their plasmids by running protein alignment against our compiled database, which includes a comprehensive pre-annotation of plasmid-encoded proteins using PlasGO.

5 Post-training: iterative fine-tuning with high-confidence pseudo-labeling

The iterative approach akin to PSI-BLAST [1] cannot be directly applied to the PlasGO model. As demonstrated in Figure 2 of the main text, during both training and prediction phases, the PlasGO model receives the same input—protein embeddings arranged in the order of their encoding in the plasmid. Notably, although the unannotated proteins' predicted probabilities do not contribute to the loss calculation due to the lack of GO annotation labels, their embedding remains a crucial input for the model. Consequently, if the parameters of the PlasGO model remain unchanged, the predicted GO probabilities for proteins will also remain constant. Incorporating the iterative search concept into our model, we designed an iterative fine-tuning strategy with high-confidence pseudo-labeling. The following Algorithm 1 is the pseudocode for this strategy:

Algorithm 1 Iterative fine-tuning with high-confidence pseudo-labeling

Data: Plasmid corpus C consisting of labeled data $L = \{protein_i, y_i\}_{i=1}^{l}$ and unlabeled data $U = \{protein_j\}_{j=1}^{u}$

Result: Converged PlasGO model p_θ

1: Initialize p_θ by training on C with only labeled data L;
2: **repeat**
3: Apply p_θ to the unlabeled data U and predict GO probabilities ($\hat{y}$) with confidence scores ($\hat{c}$), resulting in $\hat{U} = \{(protein, \hat{y}, \hat{c}) | protein \in U\}$;
4: For round r, select $\tilde{U}$ as the top $\frac{r}{10}$ of $\hat{U}$ with the highest confidence scores to serve as the pseudo-labeling data;
5: Fine tune p_θ on $L \cup \tilde{U}$ with data augmentation;
6: **until** *validation-based early stopping or reaching maximum iterations*;

Specifically, the PlasGO model undergoes initial training with 10 epochs on the original training set, aiming to produce high-quality predictions essential for serving as pseudo-labels in subsequent fine-tuning stages. Rather than employing a static threshold for selecting high-confidence pseudo-labels, the iterative fine-tuning process enables the model to dynamically learn the optimal confidence cutoff for improved prediction accuracy. Prior to the first round of fine-tuning, we employ the initialized model to predict GO probabilities and their associated confidence scores for all unannotated proteins. Then, the top 10%

(iteration number divided by 10) of the most confident predicted GO probabilities are selected as soft pseudo-labels, while the remaining 90% of unannotated labels are still masked during loss calculation. These selected soft pseudo-labels are integrated with the original training labels, and the model is fine-tuned on this augmented dataset with a lower learning rate of 5e-5 for a single epoch. In the subsequent round, the fine-tuned model from the previous iteration is used to generate pseudo-labels, with a gradual relaxation of the confidence threshold (e.g., 20% for the second round). Early stopping is employed based on the model's performance on the validation set. If there is a performance decrease in any round or the maximum of 10 rounds is reached, the iterative fine-tuning process concludes.

Supplementary Table S3. Performance comparison for PlasGO using different training methods on the RefSeq test set. The last column indicates the round at which early stopping occurred during iterative fine-tuning due to performance decrease on the validation set.

Method	GO category	F_{max}	AUPR	# of iterations
PlasGO (initial training only)	MF	0.8070	0.5165	-
	BP	0.7855	0.4638	-
	CC	0.7926	0.5109	-
PlasGO (iterative fine-tuning strategy)	MF	0.8250	0.5264	6
	BP	0.7966	0.4803	6
	CC	0.7965	0.5243	3

We assess the performance of PlasGO with the proposed iterative fine-tuning strategy against initial training alone on the RefSeq test set. The results in Supplementary Table S3 reveal that the iterative fine-tuning approach demonstrated a modest improvement across all GO categories and evaluation metrics, with the most notable enhancement of 1.8% observed in the Fmax metric within the Molecular Function (MF) category. Moreover, early stopping is activated at the sixth round for the MF and Biological Process (BP) categories. This observation indicates that the top 60% of predictions exhibit sufficient confidence and quality to be utilized as pseudo-labels for these two categories. In summary, these findings display the effectiveness of leveraging augmented datasets with pseudo-labels in enhancing GO prediction accuracy through learning plasmid patterns.

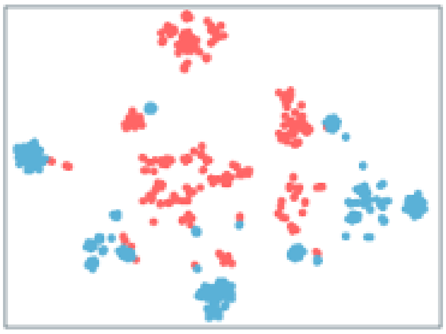

Supplementary Figure S1. The visualization of contextualized embeddings learned by PlasGO for the GO term "response to antibiotic".

To delve deeper into the reasons behind the lack of significant improvement (>2%) with the iterative fine-tuning strategy, one potential explanation could be that PlasGO has already predicted accurately for a substantial portion of samples. Consequently, the remaining misclassified samples may represent the more challenging cases. We choose Supplementary Figure S1 (a sub-figure from Supplementary Figure S6) for illustration. In this depiction, the contextualized embeddings learned by PlasGO reveal a scenario where certain positive samples (depicted by blue dots, corresponding to proteins annotated with "response to antibiotics") are intermingled with negative samples (represented by red dots, correlating to proteins without the "response to antibiotics" annotation). Despite efforts to augment our datasets with high-confidence pseudo-labels, it's still difficult to learn a clear boundary for explicitly classifying these intricate cases.

6 Rationale behind the F_{max} metric

Based on the common practice of computing F_{max}, the unannotated proteins were all excluded from the $Fmax$ metric calculation, given the absence of ground truth for evaluating prediction performance on these proteins. In line with the CAFA3 challenge [12], a recognized benchmark for validating computational tools in protein function prediction, we adhered to the standard definition of F_{max}. The original formula for this metric can be referenced in "Additional file 1" of the CAFA paper [12].

$$AvgP(\theta) = \frac{1}{m(\theta)} \cdot \sum_{i=1}^{m(\theta)} Precision_i(\theta) \tag{2}$$

To be specific, F_{max} is computed based on the probability vectors predicted by a tool and the ground-truth label encoding vectors containing binary values (0s and 1s). It is determined as the maximum F1-score achieved using arbitrary cutoffs applied to the probability vectors, varying between 0 and 1. Hence, in the formula for average precision (Equation 2), $m(\theta)$ represents the count of proteins with at least one label's predicted probability exceeding the cutoff θ, rather than the proteins with at least one annotation. For example, at a cutoff θ of 0, $m(\theta)$ equals n (the total number of test proteins). Conversely, at a very high cutoff value θ near 1, $m(\theta)$ will be lower.

For obtaining F_{max}, we compute the F1-score for each θ value within the range of 0 to 1, with an increment of 0.01, and identify the highest score as F_{max}. Empirically, the F_{max} metric reaches its peak when the cutoff θ is approximately 0.3, leading many tools to adopt 0.3 as the default probability threshold for GO term prediction. To exemplify, we utilize PlasGO's predicted probability vector within the Molecular Function (MF) category on the RefSeq test set. The variations in average precision and recall, F1-score, and the $m(\theta)$ values are delineated in Supplementary Figure S2 across the spectrum of the cutoff θ from 0 to 1.

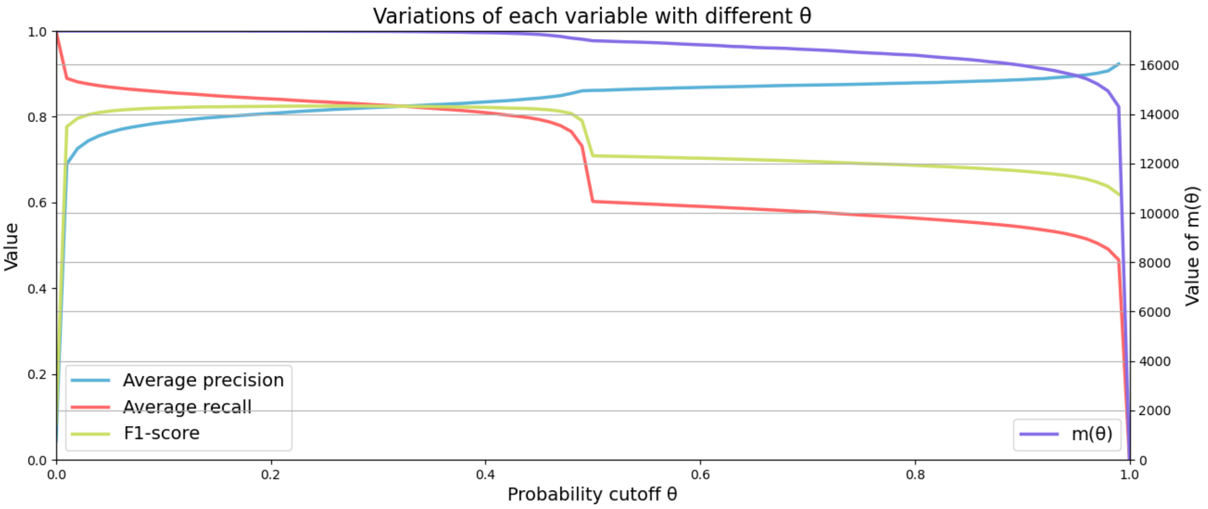

Supplementary Figure S2. The variations of average precision and recall, F1-score, $m(\theta)$ with different cutoff θ ranging from 0 to 1.

We can observe that as the cutoff θ rises, the average precision increases, while the average recall and $m(\theta)$ decline. The F1-score peaks at 0.8250 when θ equals 0.26. These findings are consistent with the above discussion.

7 Discussion comparing PlasGO and gLM

gLM is a pre-trained genomic language model [4], which integrates ESM and RoBERTa, and undergoes training on millions of metagenomic scaffolds. While gLM and PlasGO have a similar model architecture, namely a BERT family model built upon protein language models (PLMs), their design concepts and training methodologies are different. gLM functions as a transfer-learning-based foundational model designed at the genomic level (distinct from PLMs that operate at the protein level), capable of generating protein-level and contig-level embeddings for various downstream tasks [4]. The authors of gLM

conducted an experiment involving the prediction of Enzyme Commission (EC) numbers for proteins in their paper [4], a task closely related to our GO term prediction objective. Specifically, a linear probe (LP) is trained using the embeddings derived from gLM on the EC-labeled dataset. Hence, we apply the same methodology used for EC number prediction in gLM's paper to our GO term prediction tasks for comparison with PlasGO, followed by an analysis of the distinctions between the two tools.

We assessed gLM's performance on plasmid-encoded proteins through two approaches. First, we generated protein-level embeddings by feeding gLM with plasmid segments containing up to 30 proteins. Then, we trained a linear probe using gLM's contextualized embeddings on the identical dataset with PlasGO. The performance evaluation comparing PlasGO and gLM is detailed in Supplementary Table S4. Second, since gLM also functions as a foundation model, **we trained an additional version of PlasGO utilizing gLM's contextualized embeddings as input for PlasGO's core BERT module.** This benchmark follows the methodology (with the initial 10-epoch training only) outlined in the section "Ablation studies: validating PlasGO's design rationale" in the PlasGO paper. The performance comparison between PlasGO trained with ProtT5 (standard) and gLM is presented in Supplementary Table S5.

Supplementary Table S4. Performance comparison between PlasGO and the classifier based on gLM's embeddings on the RefSeq test set.

Method	GO category	F_{max}	AUPR
PlasGO	MF	0.8250	0.5264
	BP	0.7966	0.4803
	CC	0.7965	0.5243
gLM+linear probe	MF	0.4014	0.0696
	BP	0.4998	0.1501
	CC	0.6496	0.2502

Supplementary Table S5. Performance comparison between PlasGO trained upon ProtT5 and gLM on the RefSeq test set.

Pre-trained foundation model	GO category	F_{max}	AUPR
ProtT5	MF	0.8070	0.5165
	BP	0.7855	0.4638
	CC	0.7926	0.5109
gLM	MF	0.4208	0.0943
	BP	0.5332	0.1862
	CC	0.6515	0.3069

As shown in Supplementary Table S4, we can observe that the contextualized embeddings generated by gLM exhibit a weak correlation with GO annotations for plasmid-encoded proteins. This suggests that knowledge gained from the general metagenomic corpus by gLM (across all biological entities) offer limited enhancements for plasmid-specific protein tasks. Furthermore, it proves the significance of designing PlasGO, a tool specialized for protein annotation within the plasmid domain. As for the second experiment (Supplementary Table S5), while PlasGO can enhance predictions when combined with gLM as opposed to solely training a linear probe, the performance is not yet optimal. Therefore, integrating PlasGO's BERT module for capturing plasmid modular patterns with a robust PLM such as ProtTrans or ESM remains the most effective strategy.

For a more in-depth discussion, gLM's design is reasonably grounded, as a rich dataset (7.32 million sub-contigs covering a wide range of taxonomies and biological entities) can be used for its masked language modeling pretraining. However, plasmid-borne contigs constitute a very small portion of gLM's pretraining corpus, which may result in underfitting and hinder the model's ability to learn plasmid-specific genomic context information. On the other hand, although we represent plasmids as a language defined by proteins, the syntax of plasmids might differ significantly from that of other biological entities due to the specialized mechanisms of plasmids (e.g., conjugative transfer). Consequently, the knowledge gained from the general metagenomic corpus by gLM may have limited impact on improving the prediction of functions for plasmid-encoded proteins.

Another difference lies in the training methodologies employed by gLM and PlasGO. In the process of

training a linear probe for function prediction, each protein is individually trained without incorporating contextualized information, potentially leading to the loss of genomic context knowledge gained during pretraining. In contrast, PlasGO consistently undergoes training on the GO-labeled plasmid corpus, facilitating a more thorough learning and retention of contextual information to enhance functional predictions. Finally, given the complexity of GO term prediction involving a vast number of GO labels in a multi-label classification setting, we enhance PlasGO by incorporating a self-attention confidence weighting mechanism, enabling the evaluation of a confidence score for each GO prediction. As demonstrated in our paper, these confidence scores play a crucial role in generating reliable GO predictions for users.

8 McNemar's test for assessing the performance differences

Our multi-label GO term prediction on the RefSeq test set can be considered as multiple independent binary classifications across different GO labels and proteins. Given that the assumptions of normality, equal variance, and sample randomness for the tested probability vectors from each tool cannot be guaranteed, we opted for the non-parametric McNemar's test [5] to assess the statistical significance of the differences in performance between each pair of tools.

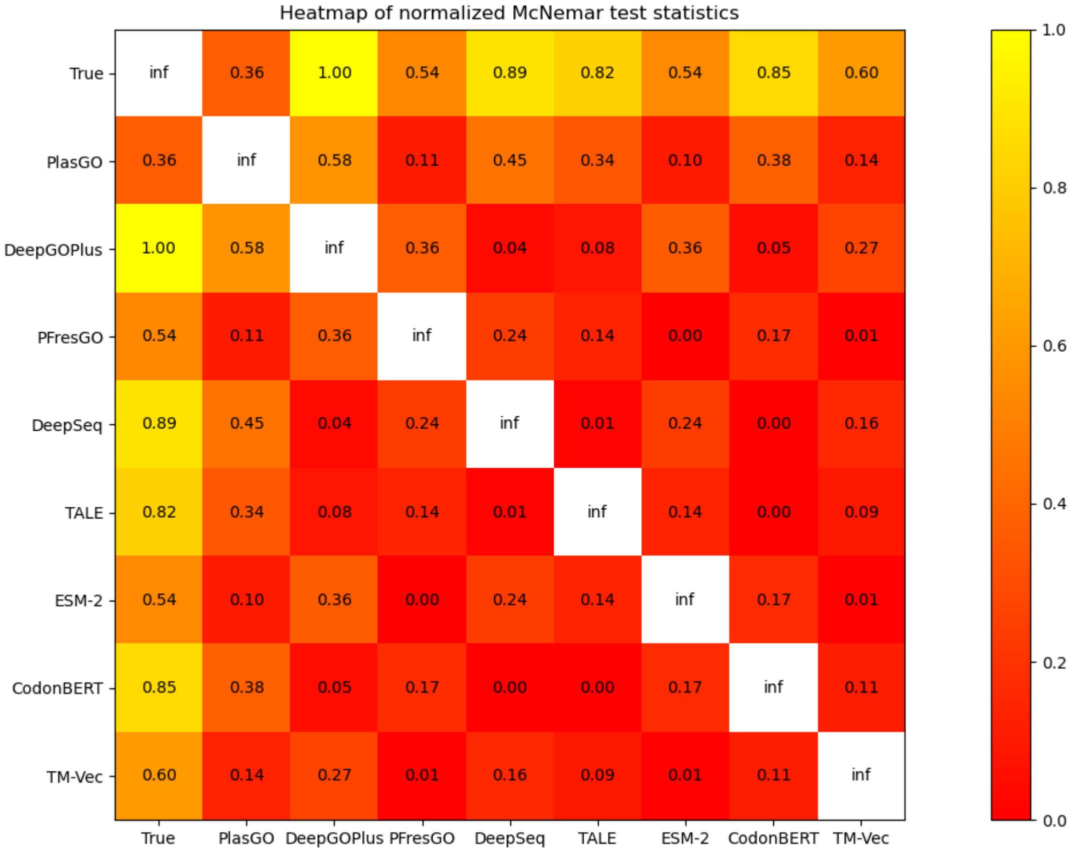

Supplementary Figure S3. The matrix illustrates the all-against-all normalized McNemar test statistics between the ground truth, PlasGO, and the seven benchmarked tools on the Molecular Function (MF) category. Each cell displays specific values, where a value close to 1 indicates a significant difference, while a value approaching 0 signifies the opposite.

Specifically, for each pair of tools, we first created a 2×2 contingency matrix comprising four values a, b, c, d. Here, b represents the count of binary classifications where the first tool is positive while the second tool is negative, and conversely, c represents the count where the first tool is negative while the second tool is positive. Then, the McNemar test statistic can be calculated as follows:

$$\chi^2 = \frac{(|b - c| - 1)^2}{b + c} \tag{3}$$

If the McNemar test statistic χ^2 is significant, we can reject the null hypothesis of equal marginal distributions and conclude that there exists a significant difference in performance between the two

tools. As depicted in Supplementary Figure S3, we computed the all-against-all normalized McNemar test statistic χ^2 between the ground truth, PlasGO, and the other seven benchmarked state-of-the-art tools on the Molecular Function (MF) category, which comprises the highest number of test proteins.

We can observe that PlasGO exhibits the lowest test statistic value compared to the ground truth in comparison to all other tools, suggesting that PlasGO performs the best on the RefSeq test set. Additionally, PlasGO consistently demonstrates a significant difference from the other tools, whereas the differences among the seven benchmarked tools are relatively minor. These statistical test results underscore the advantage of our approach in the comparative analysis.

9 Assessing PlasGO's generalizability on proteins from novel plasmid genera

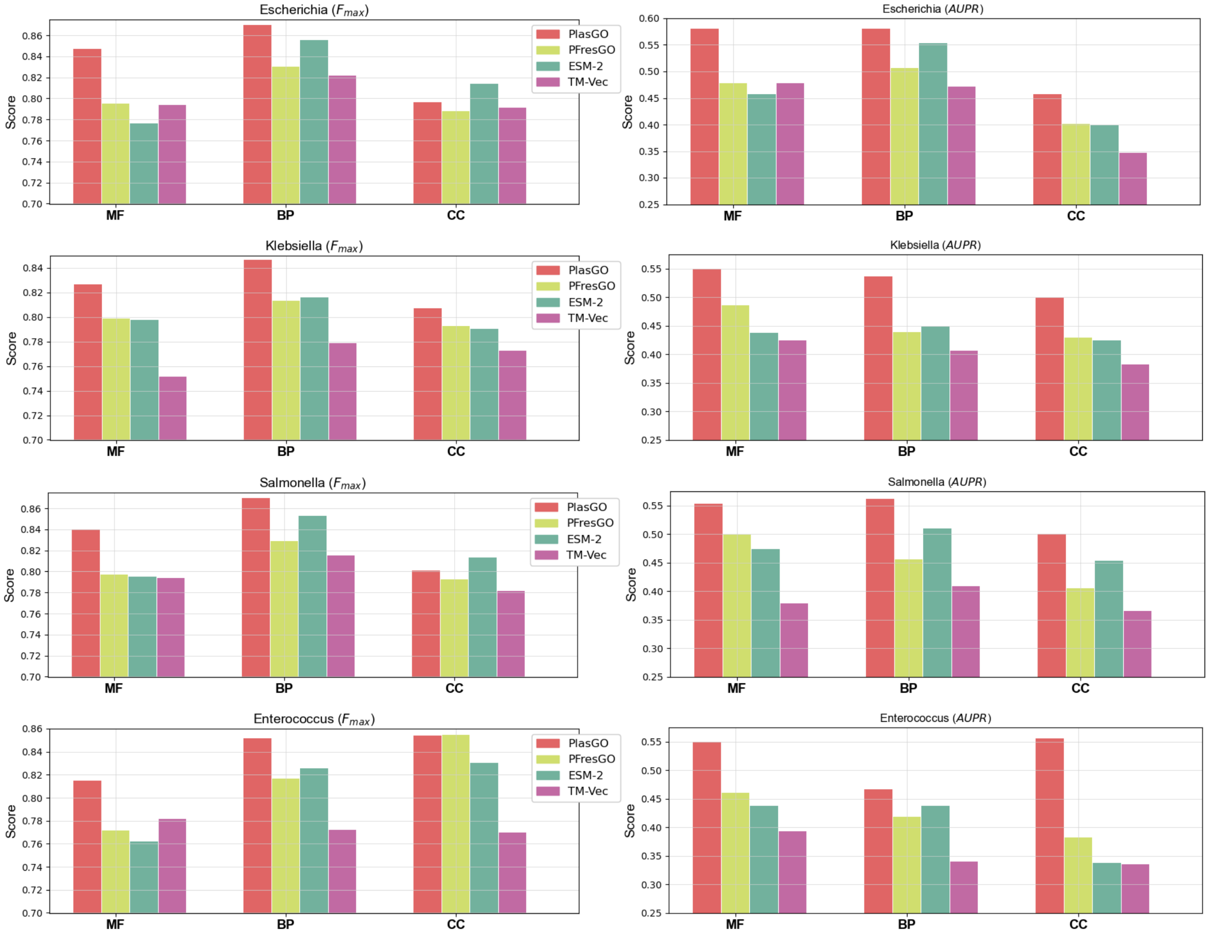

Supplementary Figure S4. The performance of PlasGO and the top three benchmarked tools on test sets derived from the four leave-one-genus-out experiment groups, evaluated using two metrics: F_{max} (left) and AUPR (right), and assessed across the three GO categories. The four rows correspond to the comparison results of proteins within the genera *Escherichia, Klebsiella, Salmonella,* and *Enterococcus*, respectively.

To evaluate PlasGO's predictive performance for proteins with plasmid taxonomy not represented in the training data, we conducted leave-one-genus-out experiments involving the top four genera with the highest plasmid counts in our RefSeq database: *Escherichia* (9,306 plasmids), *Klebsiella* (7,699 plasmids), *Salmonella* (1,999 plasmids), and *Enterococcus* (1,986 plasmids). In each experiment group, proteins with GO annotations from the selected genus were designated as the test set, while the remaining annotated proteins constituted the training and validation sets. Following the rule outlined in the "Data curation and model training" section regarding novel protein benchmark experiments, we ensured there

is no significant alignment between the training and test sets. Subsequently, PlasGO and the top three benchmarked tools were retrained for each group using the training set, and GO labels were predicted for proteins within the excluded genus in the test set. The performance evaluation measured using F_{max} and AUPR metrics, for the four tools is depicted in Supplementary Figure S4 across the four leave-one-genus-out groups.

We observed that the results of the leave-one-genus-out experiments align closely with the standard benchmarking results detailed in the "Performance on the RefSeq test set" section, with PlasGO demonstrating superior performance in terms of both F_{max} and AUPR across all three GO categories. This proves the PlasGO model's capability to accurately predict the GO-based functions of plasmid-encoded proteins, even when they originate from a genus not represented in our training data.

10 Cross-validation with plasmid-based data splitting strategy

Employing plasmid-based data splitting and cross-validation represent viable and beneficial approaches for showcasing the performance of PlasGO. Thus, we conducted a 5-fold cross-validation for PlasGO and the top three benchmarked tools. Firstly, we randomly divided all complete plasmids into five equal partitions, with each partition assigned to a fold. Within each fold, the annotated proteins from the designated partition comprised the test set, while the remaining annotated proteins constituted the training and validation sets. Aligning with the rule detailed in the "Data curation and model training" section regarding novel protein benchmark experiments, we ensured no significant alignment between the training and test sets. The performance, evaluated using F_{max} and AUPR metrics, was averaged across the five groups, and the benchmarking results are illustrated in Supplementary Figure S5.

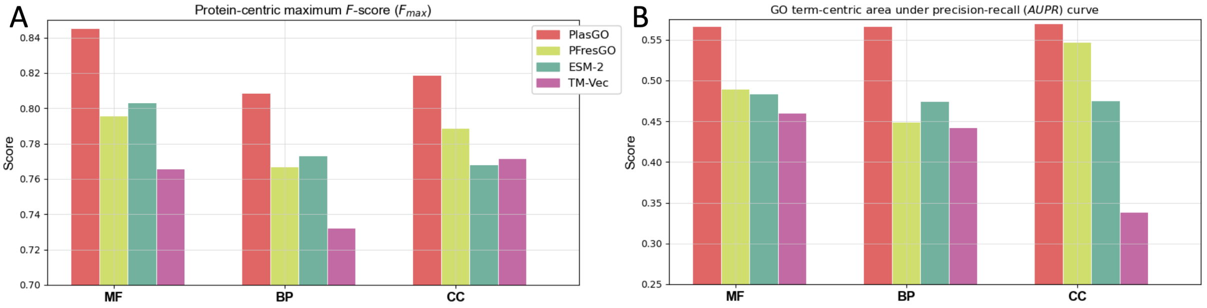

Supplementary Figure S5. The performance of PlasGO and the top three benchmarked tools averaged from the 5-fold cross-validation with plasmid-based dataset split strategy. The results are evaluated using two metrics: A) F_{max} and B) AUPR, and assessed across the three GO categories.

We can observe that the benchmark results averaged from the 5-fold cross-validation align closely with the results presented in the "Performance on the RefSeq test set" section, with PlasGO outperforming other tools in F_{max} and AUPR across all three GO categories. Compared to the single dataset split strategy, the cross-validation can provide a more reliable evaluation of PlasGO's capacity for generalization to novel proteins.

11 GO term annotations of representative plasmid core proteins

Function	Entry name	Protein name	GO terms (Molecular Function)	GO terms (Biological Process)	GO terms (Cellular Component)
Replication	TRFA_ECOLX	Plasmid replication initiator protein TrfA	GO:0003677 DNA binding	GO:0006260 DNA replication<br>GO:0006276 plasmid maintenance	GO:0005886 plasma membrane
	SSBF_ECOLI	Single-stranded DNA-binding protein	GO:0003697 single-stranded DNA binding	GO:0006260 DNA replication	
Partitioning	PARM_ECOLX	Plasmid segregation protein ParM	GO:0042802 identical protein binding	GO:0030541 plasmid partitioning	
	PARB4_ECOLX	Protein ParB	GO:0003677 DNA binding<br>GO:0004519 endonuclease activity<br>GO:0004527 exonuclease activity	GO:0030541 plasmid partitioning	GO:0005576 extracellular region
Conjugative DNA transfer	TRAI1_ECOLI	Multifunctional conjugation protein TraI	GO:0003677 DNA binding<br>GO:0003678 DNA helicase activity<br>GO:0003917 DNA topoisomerase type I (single strand cut, ATP-independent) activity<br>GO:0005524 ATP binding<br>GO:0016887 ATP hydrolysis activity<br>GO:0046872 metal ion binding	GO:0008152 metabolic process	GO:0005737 cytoplasm
	TRAC5_ECOLX	DNA primase TraC	GO:0003697 single-stranded DNA binding<br>GO:0016779 nucleotidyltransferase activity	GO:0006260 DNA replication	GO:0000428 DNA-directed RNA polymerase complex
	TRAD1_ECOLI	Coupling protein TraD	GO:0005524 ATP binding	GO:0009291 unidirectional conjugation	GO:0005886 plasma membrane
Exclusion	TRAT1_ECOLI	TraT complement resistance protein			GO:0009279 cell outer membrane
	TRAS1_ECOLI	Protein TraS			GO:0005886 plasma membrane
Type IV secretion system	PIL1_ECOLI	Pilin			GO:0005576 extracellular region<br>GO:0005886 plasma membrane
	TRAL1_ECOLI	Protein TraL		GO:0009297 pilus assembly	GO:0009279 cell outer membrane
	TRBE_RHIRD	Conjugal transfer protein TrbE	GO:0005524 ATP binding<br>GO:0016887 ATP hydrolysis activity		
	TRBL_RHIRD	Conjugal transfer protein TrbL		GO:0030255 protein secretion by the type IV secretion system	GO:0005886 plasma membrane
	TRAF_ECOLI	Protein TraF			GO:0042597 periplasmic space

12 GO term annotations of representative plasmid accessory proteins

Function	Entry name	Protein name	GO terms (Molecular Function)	GO terms (Biological Process)	GO terms (Cellular Component)
Antibiotic resistance	AADB1_KLEPN	2”-aminoglycoside nucleotidyltransferase	GO:0008871 aminoglycoside 2”-nucleotidyltransferase activity<br>GO:0046872 metal ion binding	GO:0046677 response to antibiotic	
	VANA_ENTFC	Vancomycin/teicoplanin A-type resistance protein VanA	GO:0005524 ATP binding<br>GO:0008716 D-alanine-D-alanine ligase activity<br>GO:0046872 metal ion binding	GO:0008360 regulation of cell shape<br>GO:0009252 peptidoglycan biosynthetic process<br>GO:0046677 response to antibiotic<br>GO:0071555 cell wall organization	GO:0005737 cytoplasm<br>GO:0005886 plasma membrane
Resistance to heavy metals	MERA_PSEAI	Mercuric reductase	GO:0016152 mercury (II) reductase activity<br>GO:0016668 oxidoreductase activity, acting on a sulfur group of donors, NAD(P) as acceptor<br>GO:0045340 mercury ion binding<br>GO:0050660 flavin adenine dinucleotide binding<br>GO:0050661 NADP binding	GO:0050787 detoxification of mercury ion	
	CADA1_STAAU	Cadmium-transporting ATPase	GO:0005524 ATP binding<br>GO:0008551 P-type cadmium transporter activity<br>GO:0016887 ATP hydrolysis activity<br>GO:0046872 metal ion binding	GO:0046686 response to cadmium ion	GO:0005886 plasma membrane
New metabolic process	HADB_CLODI	(R)-2-hydroxyisocaproyl-CoA dehydratase alpha subunit	GO:0016836 hydro-lyase activity<br>GO:0046872 metal ion binding<br>GO:0051539 4 iron, 4 sulfur cluster binding	GO:0006551 L-leucine metabolic process	
Virulence factors	CYAA_BACAN	Calmodulin-sensitive adenylate cyclase	GO:0004016 adenylate cyclase activity<br>GO:0005516 calmodulin binding<br>GO:0005524 ATP binding<br>GO:0008237 metallopeptidase activity<br>GO:0008294 calcium- and calmodulin-responsive adenylate cyclase activity<br>GO:0036094 small molecule binding<br>GO:0046872 metal ion binding<br>GO:0090729 toxin activity	GO:0006171 cAMP biosynthetic process<br>GO:0099004 calmodulin dependent kinase signaling pathway	GO:0005576 extracellular region<br>GO:0044164 host cell cytosol<br>GO:1902494 catalytic complex

13 Comparison results of embeddings between ProtTrans and PlasGO

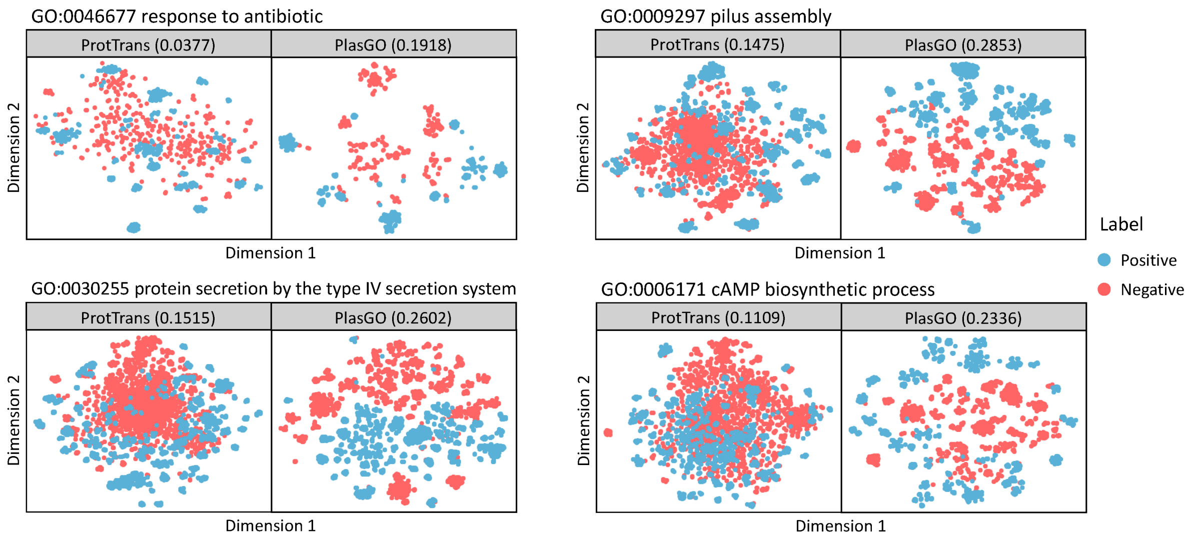

Supplementary Figure S6. Embedding comparisons for the BP binary classifications.

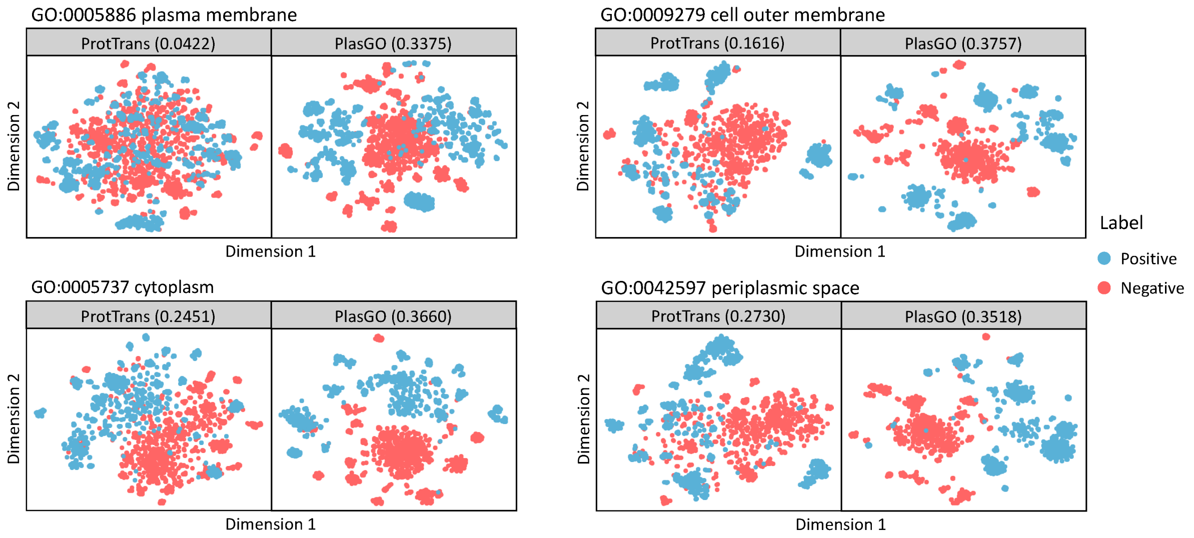

Supplementary Figure S7. Embedding comparisons for the CC binary classifications.

14 Analysis of the elusive GO term labels

Supplementary Table S6. Detailed list of the elusive labels identified using the validation set. The third column represents the AUPR scores on the validation set for the elusive labels obtained from PlasGO, all of which are below 0.3. Furthermore, the fourth to seventh columns indicate the AUPR scores on the test set for the elusive labels obtained from PlasGO, PFresGO, TM-Vec, and DeepGOPlus (the top four tools), respectively. AUPR scores on the test set that exceed 0.3 are displayed in dark red.

Category	GO term	PlasGO (val)	PlasGO	PFresGO	TM-Vec	DeepGOPlus	Detail
MF	GO:0004497	0.2303	0.8165	0.1933	0.7612	0.3892	monooxygenase activity
MF	GO:0004659	0.0331	1.0	0.0857	0.3333	0.0001	prenyltransferase activity
MF	GO:0016701	0.0338	0.0021	0.0018	0.0031	0.001	oxygenase
MF	GO:0016765	0.0673	0.0818	0.0059	0.0222	0.0014	transferase activity, transferring alkyl or aryl groups
MF	GO:0016805	0.0628	0.0321	0.0061	0.0239	0.0078	dipeptidase activity
MF	GO:0016846	0.182	0.5	0.0004	0.0092	0.0001	carbon-sulfur lyase activity
MF	GO:0019001	0.0563	0.0378	0.0541	0.041	0.0047	guanyl nucleotide binding
MF	GO:0019114	0.0928	0.1535	0.1389	0.1285	0.0012	catechol dioxygenase activity
MF	GO:0019205	0.1893	0.0757	0.1449	0.048	0.0006	nucleobase-containing compound kinase activity
MF	GO:0030145	0.1062	0.0974	0.0608	0.0805	0.0228	manganese ion binding
MF	GO:0030246	0.0227	0.2419	0.2145	0.0314	0.0055	carbohydrate binding
MF	GO:0042910	0.1166	0.0004	0.0005	0.0004	0.0006	xenobiotic transmembrane transporter activity
MF	GO:0043565	0.0474	0.0276	0.0109	0.0281	0.0168	sequence-specific DNA binding
MF	GO:0046943	0.1303	0.0036	0.0015	0.0009	0.0003	carboxylic acid transmembrane transporter activity
MF	GO:0051287	0.2041	0.1011	0.9431	0.2702	0.0232	NAD binding
MF	GO:1901682	0.0333	0.0008	0.0011	0.0009	0.0007	sulfur compound transmembrane transporter activity
BP	GO:0006081	0.0349	0.037	0.1425	0.1331	0.0352	cellular aldehyde metabolic process
BP	GO:0006457	0.2902	0.1315	0.1132	0.0764	0.008	protein folding
BP	GO:0007049	0.1268	0.0551	0.0672	0.0098	0.0088	cell cycle
BP	GO:0009605	0.1261	0.0534	0.0396	0.043	0.0315	response to external stimulus
BP	GO:0009607	0.0444	0.0435	0.0708	0.0383	0.0372	response to biotic stimulus
BP	GO:0009628	0.0179	0.25	0.0011	0.0005	0.0001	response to abiotic stimulus
BP	GO:0022402	0.0504	0.0196	0.0677	0.0084	0.0074	cell cycle process
BP	GO:0042221	0.2663	0.0552	0.0363	0.0249	0.0184	response to chemical
BP	GO:0042537	0.0963	0.3222	0.5608	0.0475	0.0093	benzene-containing compound metabolic process
BP	GO:0042592	0.1638	0.0222	0.0054	0.0133	0.0042	homeostatic process
BP	GO:0043603	0.2406	0.0332	0.0066	0.0065	0.0019	amide metabolic process
BP	GO:0044419	0.0903	0.1196	0.1367	0.0623	0.0434	interspecies interaction
BP	GO:0046451	0.0656	0.0452	0.2275	0.0181	0.0016	diaminopimelate metabolic process
BP	GO:0048518	0.0178	0.0014	0.0026	0.0125	0.0008	positive regulation of biological process
BP	GO:0048519	0.0815	0.0201	0.0225	0.0182	0.0155	negative regulation of biological process
BP	GO:0048583	0.1985	0.0001	0.0002	0.0004	0.0001	regulation of response to stimulus
BP	GO:0048878	0.1586	0.0245	0.007	0.0132	0.0042	chemical homeostasis
BP	GO:0051172	0.0791	0.0189	0.0185	0.0184	0.0155	inhibition of nitrogen metabolic process
BP	GO:0051301	0.0973	0.0761	0.0249	0.0063	0.0088	cell division
BP	GO:0080134	0.2026	0.0002	0.0001	0.0004	0.0001	regulation of response to stress
BP	GO:1901700	0.1417	0.0921	0.0408	0.0257	0.0184	response to oxygen-containing compound
CC	GO:0009986	0.242	0.025	0.0955	0.0468	0.0285	cell surface
CC	GO:0043226	0.0372	0.117	0.0629	0.0569	0.0622	organelle
CC	GO:0043229	0.0661	0.1099	0.0747	0.0462	0.0615	intracellular organelle

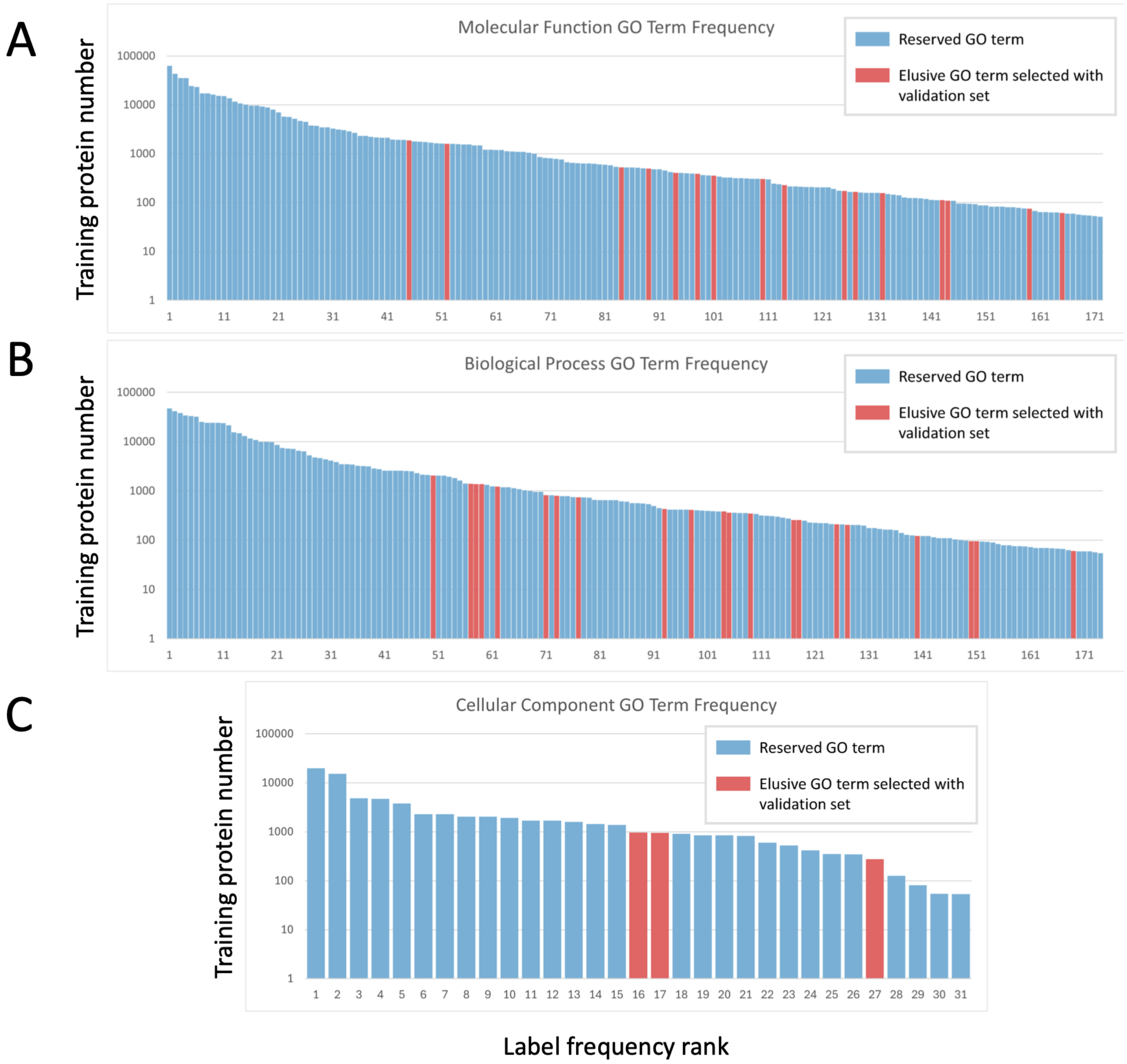

Supplementary Figure S8. Sorted occurrence frequency of GO term labels in the training set across three GO categories. The x-axis represents the ranks of the GO term labels based on their frequency, while the y-axis represents the frequency in exponential format (base 10). The red bars indicate the elusive labels, while the blue bars represent the remaining labels. It can be observed that most of the elusive labels are rare classes.

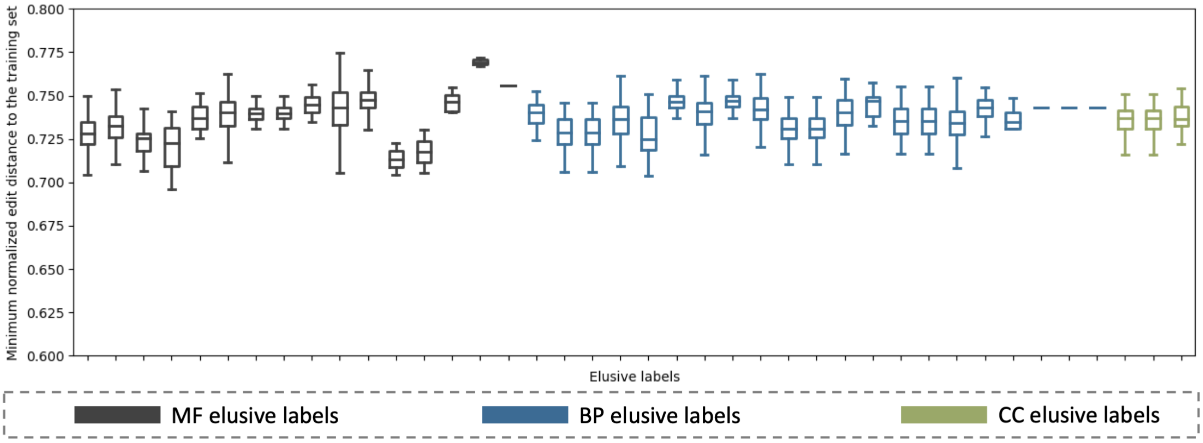

Supplementary Figure S9. The distribution of the distance between the training set and the test set for each elusive label. The distance distribution was measured by the minimum edit distance between each testing protein and the training set, normalized by dividing by the length of the longest sequence in the corresponding protein pair. The black, blue, and green boxes represent MF, BP, and CC labels, respectively. Furthermore, within each GO category, the elusive labels are sorted by their occurrence frequency in the training set. We can observe that all the minimum distances exceed 67.5%, indicating a low sequence similarity between the training set and test set for each elusive label [8].

Supplementary Table S7. The performance of different classification methods on the elusive labels evaluated using the RefSeq test set.

Method	GO category	Fmax	AUPR
3-layer DNN classifier	MF	0.1237	0.1308
	BP	0.1578	0.0449
	CC	0.735	0.0314
PlasGO (no context)	MF	0.342	0.1787
	BP	0.1578	0.0439
	CC	0.735	0.0766
PlasGO (standard)	MF	0.3773	0.1983
	BP	0.1578	0.0677
	CC	0.735	0.084

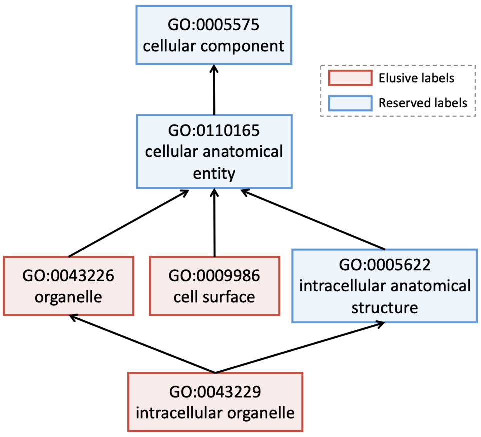

Supplementary Figure S10. The directed acyclic graph (DAG) structure including the three CC elusive labels (shown as red boxes) and their ancestor terms (shown as blue boxes).

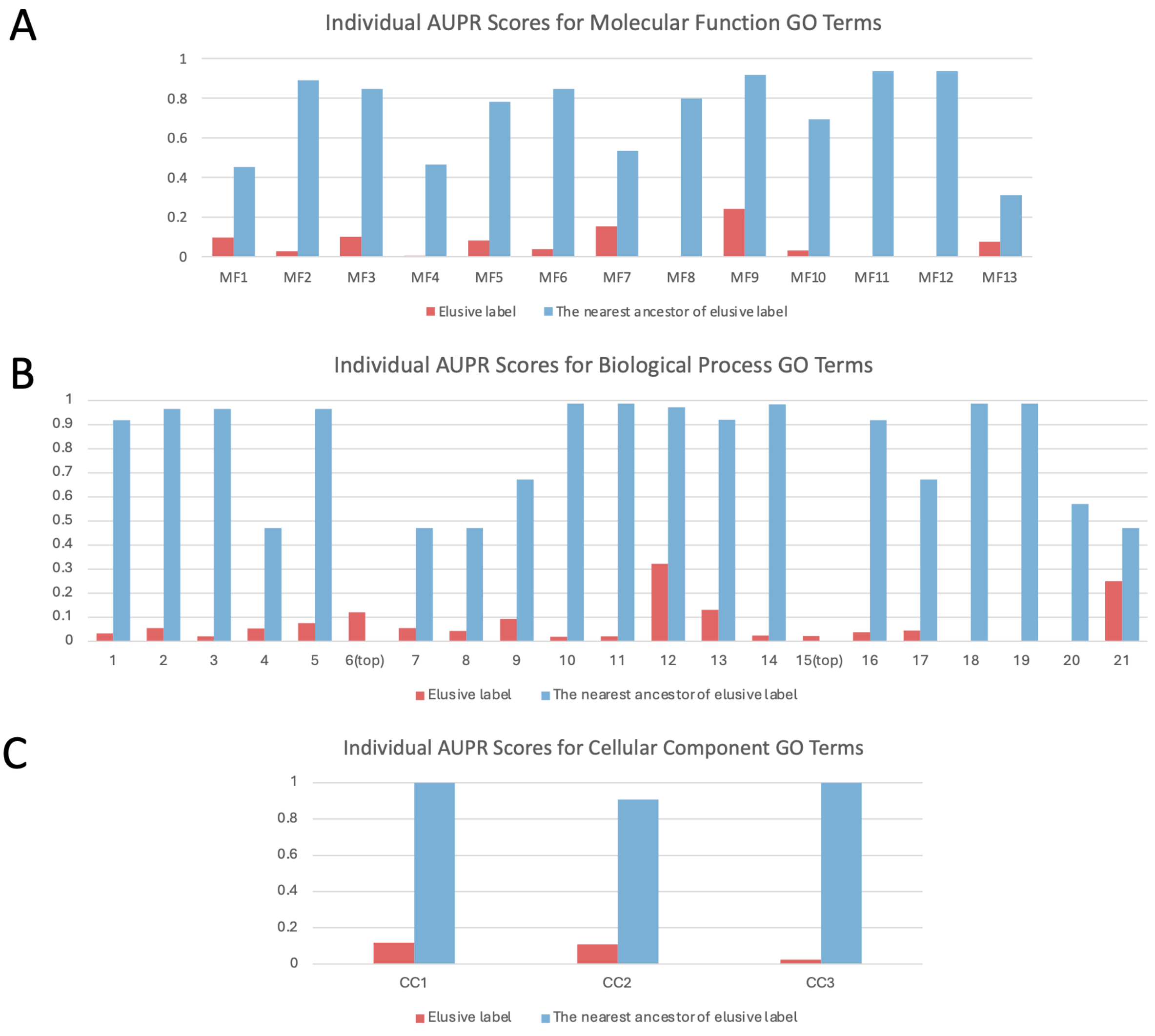

Supplementary Figure S11. The performance comparisons between the elusive labels and their nearest ancestor terms for the three GO categories. Notably, the three MF elusive labels that exhibited poor performance on the validation set but good performance on the test set, achieving AUPR scores of 0.8165, 1.0, and 0.5, respectively, are not shown. Besides, a suffix '(top)' is added to two out of the BP elusive labels, which indicates that they do not have reserved ancestor terms.

15 Evaluation of the learned confidence scores for PlasGO

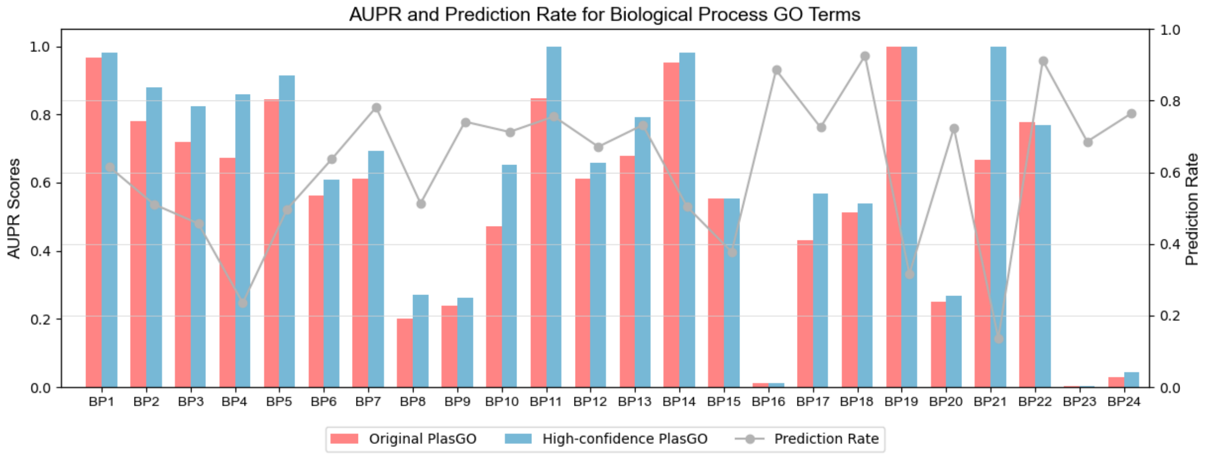

Supplementary Figure S12. The AUPR comparisons on the BP category between the original PlasGO and the high-confidence mode of PlasGO.

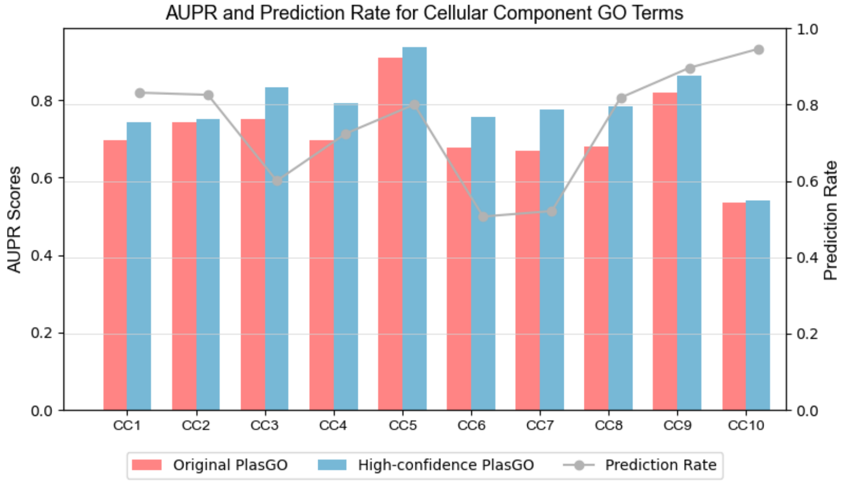

Supplementary Figure S13. The AUPR comparisons on the CC category between the original PlasGO and the high-confidence mode of PlasGO.

16 Distributions of the number of high-confidence predicted GO terms for unannotated proteins

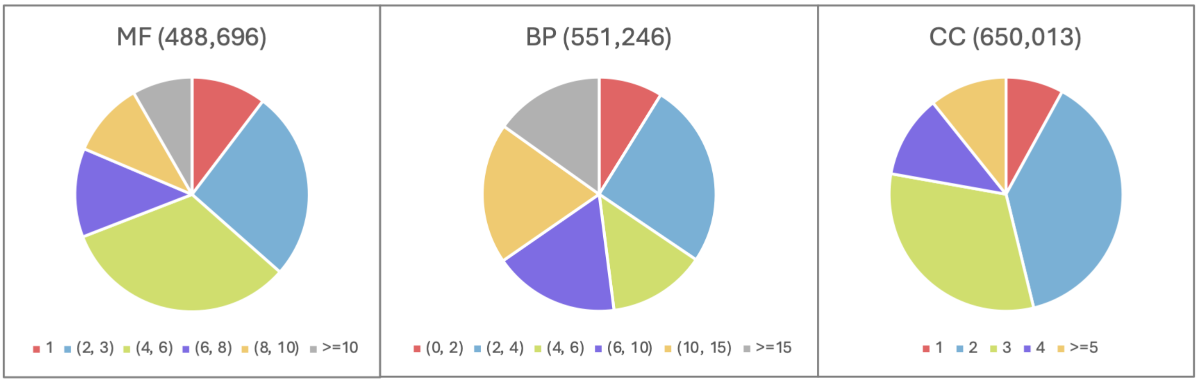

17 Comparison of PlasGO's annotation results with known biological functions

Replication is a core function of plasmids, with the origin of replication and genes encoding replication proteins constituting a minimal plasmid (also called "basic replicon") to survive within the host cell [3]. Thus, we choose to show whether our annotated GO terms can reveal the functions related to plasmid replication. To do so, we applied PlasGO to proteins collected by PlasmidFinder [2], which includes 481 replicon sequences obtained from the PCR-based replicon typing (PBRT) scheme. First, we utilize Prodigal to translate the proteins encoded within the replicon DNA sequences, yielding a total of 451 proteins. Subsequently, we employ PlasGO to predict high-confidence GO terms for these 451 proteins. Our findings are illustrated by showcasing the top 10 GO terms with the highest number of associated proteins, as depicted in Supplementary Figure S14.

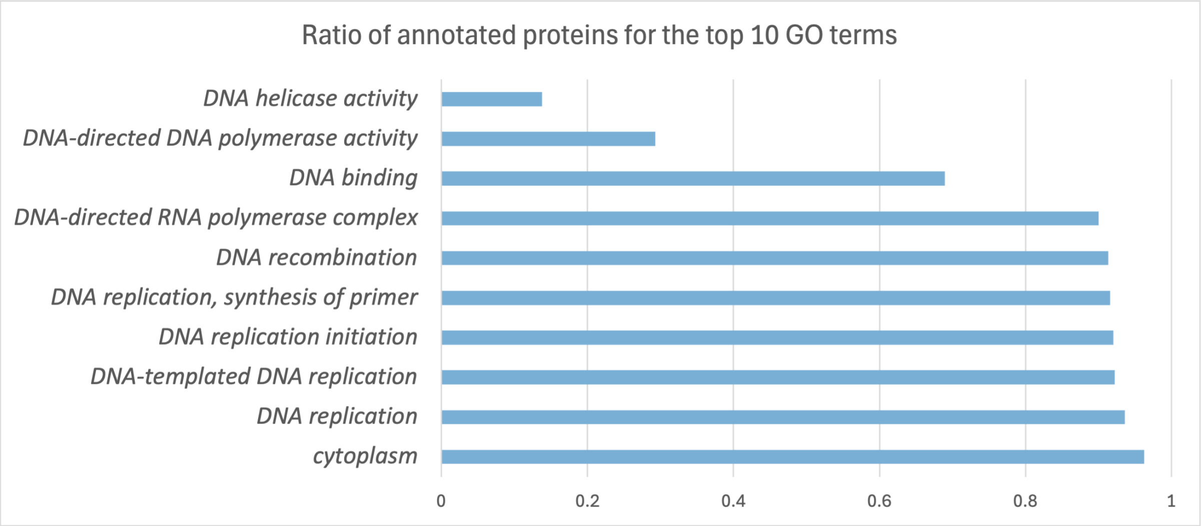

Supplementary Figure S14. The top 10 predicted GO terms with the highest number of associated proteins. Each bar represents the ratio of annotated proteins out of the total 451 proteins for each respective GO term.

We can observe that the top 10 predicted GO terms are predominantly related to plasmid replication, with the top 7 GO terms achieving a high annotation rate for the 451 proteins encoded on replicons. For instance, the two GO terms "DNA replication initiation" and "DNA helicase activity" align with the two representative proteins within the plasmid replication function category, specifically replication initiator

protein and helicase [11]. Additionally, as detailed in [10], the three plasmid replication systems rely on DNA polymerase and RNA polymerase, which can be reflected by the GO terms "DNA-directed DNA polymerase activity" and "DNA-directed RNA polymerase complex", respectively. On the other hand, in the original RefSeq database, 82.3% of the 47,871 complete plasmids encode at least one replication protein. With the expanded high-confidence GO annotations provided by PlasGO, this ratio has been increased to 98.8%. These results collectively demonstrate the strong alignment of PlasGO with known plasmid-specific biological functions.

In our study, we elucidate that the GO term indicators used to classify proteins into the three core plasmid functions (outlined in Supplementary Section S18) are semantically general (at a high level in the GO graph), enabling PlasGO to predict them with high precision. For example, PlasGO achieved a precision of 0.945 for the GO term indicator "DNA replication", as assessed on the novel RefSeq test set. Consequently, we utilize PlasGO's high-confidence mode for proteins lacking annotations, resulting in the annotation of 136,303 proteins for replication, 38,836 for conjugation, and 22,630 for stability. Moreover, PlasGO effectively assigned high-confidence GO terms to over 95% of previously unannotated proteins, demonstrating impressive precisions of 0.8229, 0.7941, and 0.8870 for the three GO categories, respectively, as evaluated on the novel RefSeq test set. These novel functional insights provided by PlasGO will serve as a valuable contribution to downstream plasmid analysis and research.

18 Complete list of the GO term indicators for the three core functions

Core function	GO term	Detail
Replication	GO:0006260	DNA replication
	GO:0003697	single-stranded DNA binding
	GO:0003678	DNA helicase activity
	GO:0006270	DNA replication initiation
	GO:0006269	DNA replication, synthesis of primer
	GO:0006261	DNA-templated DNA replication
Stability	GO:0006276	plasmid maintenance
	GO:0030541	plasmid partitioning
	GO:0007059	chromosome segregation
	GO:0051301	cell division
	GO:0110001	toxin-antitoxin complex
Conjugation	GO:0009292	horizontal gene transfer
	GO:0009297	pilus assembly
	GO:0030255	protein secretion by the type IV secretion system
	GO:0044097	secretion by the type IV secretion system
	GO:0043684	type IV secretion system complex

19 Detailed information of the proteins encoded in the two well-studied plasmids

Plasmid	Index	Protein ID	Gene product annotation	Protein class
pOLA52	1	WP_001067858	IS6-like element IS26 family transposase	MGE genes
	2	WP_000027057	broad-spectrum class A beta-lactamase TEM-1	Payload
	3	WP_000677445	type 3 fimbria minor subunit MrkF	Conjugation
	4	WP_012291466	type 3 fimbria adhesin subunit MrkD	Conjugation
	5	WP_000813718	type 3 fimbria usher protein MrkC	Conjugation
	6	WP_000820818	type 3 fimbria chaperone MrkB	Conjugation
	7	WP_002916128	type 3 fimbria major subunit MrkA	Conjugation
	8	WP_228261368	IS1-like element IS1A family transposase	MGE genes
	9	WP_001293129	H-NS family nucleoid-associated regulatory protein	Payload
	10	WP_000850859	hemolysin expression modulator Hha	Payload
	11	WP_012291470	type IA DNA topoisomerase	Replication
	12	WP_000717624	TrbM/KikA/MpfK family conjugal transfer protein	Conjugation

	13	WP_000722603	cag pathogenicity island Cag12 family protein	Payload
	14	WP_012291471	type IV secretory system conjugative DNA transfer family protein	Conjugation
	15	WP_012291472	P-type DNA transfer ATPase VirB11	Conjugation
	16	WP_012291473	VirB10/TraB/TrbI family type IV secretion system protein	Conjugation
	17	WP_000783379	TrbG/VirB9 family P-type conjugative transfer protein	Conjugation
	18	WP_000394613	type IV secretion system protein	Conjugation
	19	WP_000796673	type IV secretion system protein	Conjugation
	20	WP_000748128	EexN family lipoprotein	Conjugation
	21	WP_000744202	type IV secretion system protein	Conjugation
	22	WP_012291475	VirB3 family type IV secretion system protein	Conjugation
	23	WP_000916182	TrbC/VirB2 family protein	Conjugation
	24	WP_001446885	transcription termination/antitermination	Payload
	25	WP_000539530	MobP1 family relaxase	Conjugation
	26	WP_000757693	DNA distortion polypeptide 1	Conjugation
	27	WP_000220560	type II toxin-antitoxin system RelE/ParE family toxin	Stability
	28	WP_000121743	plasmid stabilization protein	Stability
	29	WP_001050931	RepB family plasmid replication initiator protein	Replication
	30	WP_001675596	DNA distortion polypeptide 3	Conjugation
	31	WP_012291478	ParA family protein	Stability
	32	WP_000051066	plasmid partition protein ParG	Stability
	33	WP_000864788	ParA family protein	Stability
	34	WP_000203199	recombinase family protein	Payload
	35	WP_000609146	DinQ-like type I toxin DqlB	Payload
	36	WP_272056275	DinQ-like type I toxin DqlB	Payload
	37	WP_001067858	IS6-like element IS26 family transposase	MGE genes
	38	WP_063102497	bleomycin binding protein	Payload
	39	WP_000084745	pyridoxamine 5'-phosphate oxidase family protein	Payload
	40	WP_001067858	IS6-like element IS26 family transposase	MGE genes
	41	WP_002914189	multidrug efflux RND transporter periplasmic adaptor subunit OqxA	Payload
	42	WP_000888203	Rrf2 family transcriptional regulator	Payload
pSK41	1	WP_011117677	YolD-like family protein	Payload
	2	WP_001273859	recombinase family protein	Payload
	3	WP_000331763	ArdC family protein	Stability
	4	WP_001252101	MobA/MobL family protein	Conjugation
	5	WP_001819633	parM protein	Stability
	6	WP_000358311	recombinase	Payload
	7	WP_001008213	helix-turn-helix transcriptional regulator	Payload
	8	WP_000043161	replication initiator protein A	Replication
	9	WP_001106022	IS6-like element IS257 family transposase	MGE genes
	10	WP_102695945	protein rep	Replication
	11	WP_012695373	sulfite exporter TauE/SafE family protein	Payload
	12	WP_001106022	IS6-like element IS257 family transposase	MGE genes
	13	WP_227992126	protein rep	Replication
	14	WP_000119405	MobV family relaxase	Conjugation
	15	WP_001242578	bleomycin binding protein	Payload
	16	WP_001795128	aminoglycoside O-nucleotidyltransferase ANT(4')-Ia	Payload
	17	WP_001106019	IS6-like element IS257 family transposase	MGE genes
	18	WP_000368849	conjugative transfer protein TrsA	Conjugation
	19	WP_000591622	CagC family type IV secretion system protein	Conjugation
	20	WP_000979860	TrsD/TraD family conjugative transfer protein	Conjugation
	21	WP_000735569	TrsH/TraH family protein	Conjugation
	22	WP_001094109	DNA topoisomerase III	Replication
	23	WP_000209436	type IV secretory system conjugative DNA transfer family protein	Conjugation
	24	WP_011117679	conjugal transfer protein TrbL family protein	Conjugation
	25	WP_000608970	single-stranded DNA-binding protein	Replication
	26	WP_011117680	IS6-like element IS257 family transposase	MGE genes
	27	WP_001579520	protein rep	Replication
	28	WP_001146389	quaternary ammonium compound efflux SMR transporter QacC	Payload
	29	WP_001105984	IS6-like element IS257 family transposase	MGE genes
	30	WP_000393259	GNAT family N-acetyltransferase	Payload
	31	WP_001028144	aminoglycoside O-phosphotransferase APH(2")-Ia	Payload
	32	WP_223200496	IS6 family transposase	MGE genes
	33	WP_000889978	type I toxin-antitoxin system Fst family toxin	Stability

	34	WP_001106006	IS6-like element IS257 family transposase	MGE genes

20 Comparison of GO annotations for plasmid pSK41 between the raw RefSeq database and the predictions generated by PlasGO

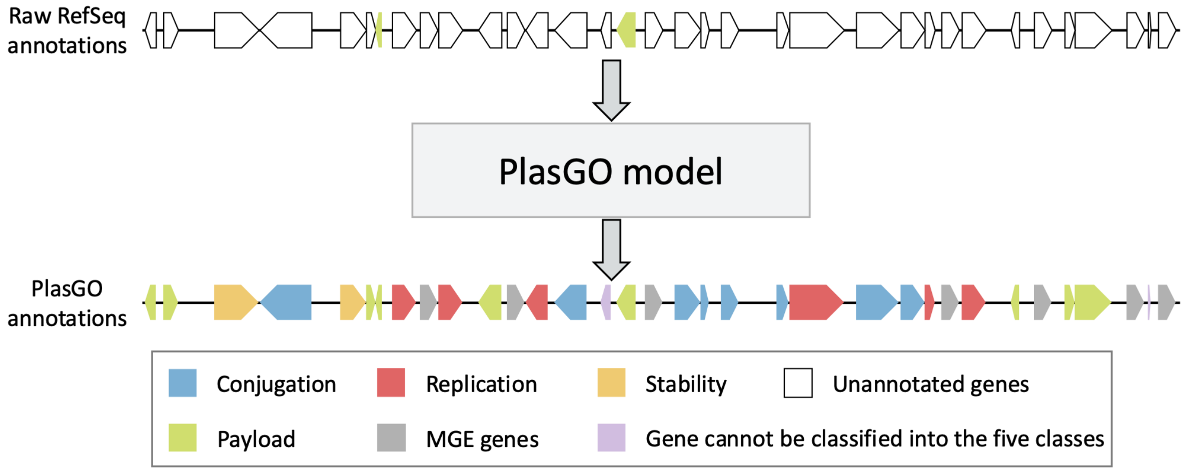

Supplementary Figure S15. Comparison of GO annotations for proteins encoded on plasmid pSK41 between the raw RefSeq database (above) and predictions generated by PlasGO (below). The proteins are classified into five functional classes using the respective GO term indicators. We can observe that the raw RefSeq database contains GO annotations for only two proteins within the payload functional class. In contrast, PlasGO effectively assigned GO annotations to all proteins encoded on plasmid pSK41, with the exception of two proteins (highlighted by purple pentagon blocks) that could not be categorized into the five functional classes using the GO term indicators.

References

[1] Stephen F Altschul, Thomas L Madden, Alejandro A Schäffer, Jinghui Zhang, Zheng Zhang, Webb Miller, and David J Lipman. Gapped blast and psi-blast: a new generation of protein database search programs. *Nucleic acids research*, 25(17):3389–3402, 1997.

[2] Alessandra Carattoli and Henrik Hasman. Plasmidfinder and in silico pmlst: identification and typing of plasmid replicons in whole-genome sequencing (wgs). *Horizontal gene transfer: methods and protocols*, pages 285–294, 2020.

[3] Ian Dewan and Hildegard Uecker. A mathematician's guide to plasmids: an introduction to plasmid biology for modellers. *Microbiology*, 169(7):001362, 2023.

[4] Yunha Hwang, Andre L Cornman, Elizabeth H Kellogg, Sergey Ovchinnikov, and Peter R Girguis. Genomic language model predicts protein co-regulation and function. *Nature communications*, 15(1):2880, 2024.

[5] Nathalie Japkowicz and Mohak Shah. *Evaluating learning algorithms: a classification perspective.* Cambridge University Press, 2011.

[6] Joshua Meier, Roshan Rao, Robert Verkuil, Jason Liu, Tom Sercu, and Alex Rives. Language models enable zero-shot prediction of the effects of mutations on protein function. *Advances in neural information processing systems*, 34:29287–29303, 2021.

[7] Tong Pan, Chen Li, Yue Bi, Zhikang Wang, Robin B Gasser, Anthony W Purcell, Tatsuya Akutsu, Geoffrey I Webb, Seiya Imoto, and Jiangning Song. Pfresgo: an attention mechanism-based deep-learning approach for protein annotation by integrating gene ontology inter-relationships. *Bioinformatics*, 39(3):btad094, 2023.

[8] Burkhard Rost. Twilight zone of protein sequence alignments. *Protein engineering*, 12(2):85–94, 1999.

[9] Jiayu Shang, Cheng Peng, Yongxin Ji, Jiaojiao Guan, Dehan Cai, Xubo Tang, and Yanni Sun. Accurate and efficient protein embedding using multi-teacher distillation learning. *Bioinformatics*, 40(9):btae567, 2024.

[10] Masaki Shintani and Haruo Suzuki. Plasmids and their hosts. *DNA Traffic in the Environment*, pages 109–133, 2019.

[11] Christopher M Thomas, Nicholas R Thomson, Ana M Cerdeño-Tárraga, Celeste J Brown, Eva M Top, and Laura S Frost. Annotation of plasmid genes. *Plasmid*, 91:61–67, 2017.

[12] Naihui Zhou, Yuxiang Jiang, Timothy R Bergquist, Alexandra J Lee, Balint Z Kacsoh, Alex W Crocker, Kimberley A Lewis, George Georghiou, Huy N Nguyen, Md Nafiz Hamid, et al. The cafa challenge reports improved protein function prediction and new functional annotations for hundreds of genes through experimental screens. *Genome biology*, 20:1–23, 2019.
