# Supplementary material for: PlasGO: enhancing GO-based function prediction for plasmid-encoded proteins based on genetic structure
Source: Gigascience. 2024 Dec 20;13:giae104. doi: 10.1093/gigascience/giae104 (PMC11659980; doi:10.1093/gigascience/giae104)
Supplement: giae104_Response_to_Reviewer_Comments_Original_Submission [file giae104_response_to_reviewer_comments_original_submission.pdf]

Author's Response To Reviewer Comments

Dear Editor and Reviewers,
We would like to thank you for your careful reading of our paper and the valuable comments. We have addressed the comments of all reviewers, with their comments in italic and our responses in blue. The revised figures and descriptions were added to the main manuscript and the supplementary file. The changes in the main draft are highlighted in red. For a more easily readable PDF version of the response letter containing inserted figures and tables, please refer to the attached “Response letter.pdf” file.

Before we provide the detailed responses, we would like to summarize the major improvements or modifications. Due to the page limit, new results were mainly added to the Supplementary file.
1. Inspired by iterative protein search tools, we devised a post-training phase featuring an iterative fine-tuning strategy based on high-confidence pseudo-labeling. Specifically, a subset of the most confidently predicted Gene Ontology (GO) probabilities (selected with a gradually relaxing confidence threshold across iterations) from one round of fine-tuning will be utilized as additional soft pseudo labels for the next round. Experimental results demonstrated a modest improvement in post-training performance compared to traditional training relying solely on labeled data.
2. To make our benchmarking more convincing, we incorporated two additional large language model (LLM)-based state-of-the-art tools, ESM-2 and CodonBERT, both of which can be optimized for predicting the GO annotations of plasmid-encoded proteins.
3. We explored the difference between gLM and PlasGO through method analysis and benchmarking experiments. gLM, a pre-trained genomic language model, combining protein language model (PLM) and a BERT family model, undergoing training on millions of metagenomic scaffolds. While gLM and PlasGO share a similar model architecture, their design concepts and training approaches diverge. Benchmarking results revealed that gLM’s contextualized embeddings demonstrate a weak correlation with the GO-based functions of plasmid-encoded proteins. This suggests that the knowledge gained from the general metagenomic corpus (across a wide range of biological entities) by gLM offers limited assistance for plasmid-specific protein tasks. Furthermore, it highlights the novelty and effectiveness of our training methodology, where the model is consistently trained on a contextual corpus.
4. To further assess PlasGO’s ability to generalize to novel proteins, including those from plasmid taxonomies that are not present in our training data, we performed a 5-fold cross-validation and four groups of leave-one-genus-out benchmarking experiments.
5. We substituted the asymmetrical metric with the symmetrical Jaccard index for the case study involving two well-studied conjugative plasmids.
6. To illustrate how our results align with established biological functions, we compared the function predictions by PlasGO with the annotations from two popular alignment-based plasmid typing tools, PlasmidFinder and CONJscan. These tools can be employed for annotating proteins in the replication and conjugation classes, respectively.
7. We enriched our paper with discussions and experiments covering several aspects of the PlasGO method. These encompass the limitations of PlasGO (the presence of elusive GO labels) and potential methods for improvement, the rationale behind selecting the RefSeq protein database, the flexibility of PlasGO for predicting long proteins, the incorporation of statistical significance tests for benchmarking results, the computational costs and resource requirements for PlasGO, strategies employed to prevent overfitting, and the integration of PlasGO into existing bioinformatics workflows.

Authors’ response
Reviewer 1:
1. The manuscript introduces PlasGO, which leverages a hierarchical architecture for GO term prediction in plasmid-encoded proteins. However, the novelty of the approach could be questioned. While the combination of protein language models and BERT for GO prediction is innovative, similar methods have been applied in other contexts.
Thank you. We acknowledge that PlasGO is not the only method to combine BERT family models with protein language models (PLMs). Given the great success of language models, more and more research projects have adopted this idea. However, based on our tests, none of the available methods can do a satisfactory job for our research problem. Below we summarize the novel parts that make PlasGO an

optimal choice for Gene Ontology (GO) prediction in plasmid sequences. PlasGO's three-layer hierarchical architecture is optimized specifically for the GO prediction of plasmid-encoded proteins. Within the model, the local PLM is utilized to generate meaningful embeddings for proteins, while the global BERT module captures plasmid modular patterns to enhance functional predictions for each protein. Additionally, PlasGO incorporates a self-attention confidence weighting mechanism, which evaluates a confidence score for each GO prediction. The experimental results demonstrate the pivotal roles played by all three components of the PlasGO method in achieving commendable performance in the intricate multi-label GO prediction task.
For a deeper exploration of the novelty of our approach, we have chosen gLM [1] for comparative analysis. gLM is a pre-trained genomic language model, which integrates ESM (a state-of-the-art PLM) and RoBERTa (a variant of BERT), and undergoes training on millions of metagenomic scaffolds. While gLM and PlasGO share a similar model architecture, their design concepts and training methodologies differ a lot. gLM functions as a transfer-learning-based foundational model designed on a different level from PLM, capable of generating protein-level and contig-level embeddings for various downstream tasks. Its design is reasonably grounded, as a rich dataset (7.32 million sub-contigs covering a wide range of taxonomies and biological entities) can be used for its masked language modeling pretraining. However, plasmid-borne contigs constitute a very small portion of gLM's pretraining corpus, which may result in underfitting and hinder the model's ability to learn plasmid-specific genomic context information. On the other hand, although we represent plasmids as a language defined by proteins, the syntax of plasmids might differ significantly from that of other biological entities due to the specialized mechanisms of plasmids (e.g., conjugative transfer). Thus, it remains uncertain whether knowledge gained from the general metagenomic corpus by gLM can effectively help in predicting the functions of plasmid-encoded proteins. This uncertainty prompted us to conduct a series of experiments, which will be shown below.
Another difference lies in the training methodologies employed by gLM and PlasGO. The authors of gLM conducted an experiment involving the prediction of Enzyme Commission (EC) numbers for proteins in their paper [1], a task closely related to our GO term prediction objective. Specifically, a linear probe (LP) is trained using the embeddings derived from gLM on the EC-labeled dataset. In this process, each protein is individually trained without incorporating contextualized information, potentially leading to the loss of genomic context knowledge gained during pretraining. In contrast, PlasGO consistently undergoes training on the GO-labeled plasmid corpus, facilitating a more thorough learning and retention of contextual information to enhance functional predictions.
We assessed gLM's performance on plasmid-encoded proteins through two approaches. First, aligning with the method employed in gLM's paper for predicting EC numbers, we generated protein-level embeddings by feeding gLM with plasmid segments containing up to 30 proteins. Subsequently, we trained a linear probe using gLM's contextualized embeddings on the identical dataset with PlasGO. The performance evaluation comparing PlasGO and gLM is detailed in Supplementary Table S4.
We can observe that the contextualized embeddings generated by gLM exhibit a weak correlation with GO annotations for plasmid-encoded proteins. This suggests that knowledge gained from the general metagenomic corpus (across all biological entities) offer limited enhancements for plasmid-specific protein tasks. Furthermore, it proves the significance of designing PlasGO, a tool specialized for protein annotation within the plasmid domain.
Next, since gLM functions as a foundation model similar to PLMs, we trained an additional version of PlasGO utilizing gLM's contextualized embeddings as input for PlasGO's core BERT module. This benchmark follows the methodology outlined in the section "Ablation studies: validating PlasGO's design rationale" in the PlasGO paper. The performance comparison between PlasGO trained with ProtT5 (standard) and gLM is presented in Supplementary Table S5.
While PlasGO can enhance predictions when combined with gLM as opposed to solely training a linear probe, the performance is not yet optimal. Therefore, integrating PlasGO's BERT module for capturing plasmid modular patterns with a robust PLM such as ProtTrans or ESM remains the most effective strategy.
The above experiments and discussions comparing PlasGO and gLM are mentioned in the "SOTA tools for benchmarking" section and elaborated in Supplementary Section S7.

2. The study heavily relies on data from the RefSeq database, yet there is limited discussion on the quality and completeness of this data. The manuscript should address potential biases introduced by incomplete or incorrect GO annotations in the database. Moreover, the study uses protein sequences of up to 1K length, which might exclude relevant longer sequences, potentially limiting the model's applicability to all plasmid-encoded proteins.
Thanks. We chose to conduct our experiments using RefSeq due to the rigorous quality assurance (QA)

checks administered by NCBI staff on all data within RefSeq before its public release [2]. This meticulous process results in high-quality RefSeq protein databases that significantly mitigate the occurrence of incorrect GO annotations. Moreover, the RefSeq database provides access to the genomic context information, specifically the order in which proteins are encoded in the plasmid, a key feature for PlasGO that is not available in other protein-only databases like Swiss-Prot. On the other hand, despite the long-standing issue of incompleteness in the GO annotation domain, several previous studies have demonstrated the meaningfulness and reliability of current large-scale GO evaluations [3][4]. As GO annotations continue to expand over time, the problem of incompleteness is expected to gradually improve. In light of these considerations, we opted for the use of the more accurate RefSeq database in developing our tool, as it allows our model to better capture the distinctive features of plasmid-encoded proteins with reduced noise and misinterpretation. The above discussions have also been included as the first paragraph in the “RefSeq dataset” section.
We restricted the maximum protein length to 1Kbp for training PlasGO because this limit is computationally efficient for the Transformer architecture, a common practice followed by many state-of-the-art protein-related methods such as ESM [5] and PFresGO [6]. Another reason is that the average length of all RefSeq plasmid-encoded proteins is 293bp, with only 2.18% of them larger than 1Kbp, indicating that removing these larger proteins would not result in a significant loss of features for PlasGO to learn. Nonetheless, in the prediction phase or when utilizing our PlasGO tool, no length restrictions are imposed. Therefore, PlasGO can accept users’ input proteins exceeding 1K in length and predict their functions drawing from the knowledge acquired from shorter proteins. To evaluate this capability, we conducted an experiment where we predicted GO terms for the larger proteins without further training or fine-tuning. The performance comparison between PlasGO and the other top three tools is presented in Supplementary Table S1. PlasGO exhibits the best overall performance on proteins larger than 1Kbp. This indicates the potential for our method to generalize to plasmid-encoded proteins of varying lengths. The investigation into the protein length limit as described above is also mentioned in the “RefSeq dataset” section and detailed in Supplementary Section S2.

3. The manuscript claims that PlasGO can generalize well to novel proteins, but this claim is based on a specific dataset. The model's generalizability should be tested on more diverse and independent datasets, including plasmids from different bacterial species or environmental contexts.
Thanks for this suggestion. To evaluate PlasGO’s predictive performance for proteins with plasmid taxonomy not represented in the training data, we conducted leave-one-genus-out experiments involving the top four genera with the highest plasmid counts in our RefSeq database: Escherichia (9,306 plasmids), Klebsiella (7,699 plasmids), Salmonella (1,999 plasmids), and Enterococcus (1,986 plasmids). In each experiment group, proteins with GO annotations from the selected genus were designated as the test set, while the remaining annotated proteins constituted the training and validation sets. Following the rule outlined in the “Data curation and model training” section regarding novel protein benchmark experiments, we ensured there is no significant alignment between the training and test sets. Subsequently, PlasGO and the top three benchmarked tools were retrained for each group using the training set, and GO labels were predicted for proteins within the excluded genus in the test set. The performance evaluation measured using Fmax and AUPR metrics, for the four tools is depicted in Supplementary Figure S4 across the four leave-one-genus-out groups.
We observed that the results of the leave-one-genus-out experiments align closely with the standard benchmarking results detailed in the “Performance on the RefSeq test set” section, with PlasGO demonstrating superior performance in terms of both Fmax and AUPR across all three GO categories. This proves the PlasGO model’s capability to accurately predict the GO-based functions of plasmid-encoded proteins, even when they originate from a genus not represented in our training data. This experiment has been outlined in the “Performance on the RefSeq test set” section and detailed in Supplementary Section S9.

4. While the model's performance is quantitatively evaluated, the interpretability of the results remains unclear. The study should include an analysis of how well the model's predictions align with known biological functions and pathways. Additionally, it would be helpful to include examples where PlasGO provides novel insights that other models do not, thereby demonstrating its practical utility.
We thank the reviewer for the comment. Replication is a core function of plasmids, with the origin of replication and genes encoding replication proteins constituting a minimal plasmid (also called “basic replicon”) to survive within the host cell [7]. Thus, we choose to show whether our annotated GO terms

can reveal the functions related to plasmid replication. To do so, we applied PlasGO to proteins collected by PlasmidFinder [8], which includes 481 replicon sequences obtained from the PCR-based replicon typing (PBRT) scheme. First, we utilize Prodigal to translate the proteins encoded within the replicon DNA sequences, yielding a total of 451 proteins. Subsequently, we employ PlasGO to predict high-confidence GO terms for these 451 proteins. Our findings are illustrated by showcasing the top 10 GO terms with the highest number of associated proteins, as depicted in Supplementary Figure S14.
We can observe that the top 10 predicted GO terms are predominantly related to plasmid replication, with the top 7 GO terms achieving a high annotation rate for the 451 proteins encoded on replicons. For instance, the two GO terms "DNA replication initiation" and "DNA helicase activity" align with the two representative proteins within the plasmid replication function category, specifically replication initiator protein and helicase [9]. Additionally, as detailed in [10], the three plasmid replication systems rely on DNA polymerase and RNA polymerase, which can be reflected by the GO terms "DNA-directed DNA polymerase activity" and "DNA-directed RNA polymerase complex", respectively. On the other hand, in the original RefSeq database, 82.3% of the 47,871 complete plasmids encode at least one replication protein. With the expanded high-confidence GO annotations provided by PlasGO, this ratio has been increased to 98.8%. These results collectively demonstrate the strong alignment of PlasGO with known plasmid-specific biological functions.
In our study, we elucidate that the GO term indicators used to classify proteins into the three core plasmid functions (outlined in Supplementary Section S18) are semantically general (at a high level in the GO graph), enabling PlasGO to predict them with high precision. For example, PlasGO achieved a precision of 0.945 for the GO term indicator "DNA replication", as assessed on the novel RefSeq test set. Consequently, we utilize PlasGO's high-confidence mode for proteins lacking annotations, resulting in the annotation of 136,303 proteins for replication, 38,836 for conjugation, and 22,630 for stability. Moreover, PlasGO effectively assigned high-confidence GO terms to over 95% of previously unannotated proteins, demonstrating impressive precisions of 0.8229, 0.7941, and 0.8870 for the three GO categories, respectively, as evaluated on the novel RefSeq test set. These novel functional insights provided by PlasGO will serve as a valuable contribution to downstream plasmid analysis and research.
The above experiments and discussions have also been outlined in the "Application: automatic GO prediction for unannotated plasmid-encoded proteins in RefSeq" section and elaborated in Supplementary Section S17.

5. The manuscript does not provide detailed information on the computational resources required to train and run PlasGO. Given the complexity of the model, there are potential concerns about its scalability, particularly for larger plasmid datasets or in settings with limited computational power.
We thank the reviewer for the comment. The PlasGO model is structured as a flexible architecture, featuring a core BERT module integrated with raw per-protein embeddings from various protein language models (PLMs), including models from the ProtTrans family and the ESM family. As demonstrated in the "Ablation studies" experiment section, PlasGO achieves robust performance across a spectrum of PLMs with varying parameter counts. Hence, we tested the maximum GPU memory usage and the runtime for both the training and prediction phases of PlasGO built using the standard ProtT5 model and three alternatives, more lightweight ESM models.
Specifically, each group was trained with a batch size of 32 for 10 epochs. For the prediction phase, we randomly chose 1,000 test plasmids encoding 72,634 proteins. Both the training and prediction involved two steps: the initial preprocessing step to extract per-protein embeddings with PLM, followed by the second step to run the PlasGO model. Notably, all experiments were conducted on a single NVIDIA GeForce RTX 3090 Blower 24G graphics card.
As shown in Supplementary Table S2, we can conclude that regardless of the employed PLMs, PlasGO requires minimal computational resources, particularly during the prediction phase. This efficiency stems from PlasGO's core module, structured as a lightweight BERT model with a fixed hidden size of 512 and a small number of Transformer layers (2 for the Cellular Component category and 4 for the other two). Conversely, the preprocessing step could require increased computational resources when employing a large PLM. Since we have provided all training codes, users can readily train their custom models using PLMs that align with their computational capabilities. For instance, users with limited GPU memory could opt for an ESM model with 8 or 35 million parameters to integrate with PlasGO. Additionally, leveraging a knowledge distillation-based protein embedding method, such as MTDP [11], offers a good alternative that significantly reduces resource demands while maintaining comparable performance. Finally, for users without a GPU, they can still annotate their plasmids by running protein alignment against our compiled

database, which includes a comprehensive pre-annotation of plasmid-encoded proteins using PlasGO.
The above evaluation concerning the computational costs and resource requirements of PlasGO has been referenced in the “PlasGO model training” section and elaborated in Supplementary Section S4.

6. The manuscript compares PlasGO with several state-of-the-art tools, but the comparison might not be fully exhaustive. Additionally, statistical significance tests for performance differences should be provided to support the comparative analysis.
We appreciate the reviewer for this valuable suggestion. Our multi-label GO term prediction on the RefSeq test set can be considered as multiple independent binary classifications across different GO labels and proteins. Given that the assumptions of normality, equal variance, and sample randomness for the tested probability vectors from each tool cannot be guaranteed, we opted for the non-parametric McNemar’s test [12] to assess the statistical significance of the differences in performance between each pair of tools. Specifically, for each pair of tools, we first created a 2×2 contingency matrix comprising four values a, b, c, d. Here, b represents the count of binary classifications where the first tool is positive while the second tool is negative, and conversely, c represents the count where the first tool is negative while the second tool is positive. Then, the McNemar test statistic can be calculated as follows (Supplementary Equation S3):
$$\chi^2 = \frac{(|b-c|-1)^2}{b+c}$$
If the McNemar test statistic χ^2 is significant, we can reject the null hypothesis of equal marginal distributions and conclude that there exists a significant difference in performance between the two tools. As depicted in Supplementary Figure S3, we computed the all-against-all normalized McNemar test statistic χ^2 between the ground truth, PlasGO, and the other seven benchmarked state-of-the-art tools on the Molecular Function (MF) category, which comprises the highest number of test proteins.
We can observe that PlasGO exhibits the lowest test statistic value compared to the ground truth in comparison to all other tools, suggesting that PlasGO performs the best on the RefSeq test set. Additionally, PlasGO consistently demonstrates a significant difference from the other tools, whereas the differences among the seven benchmarked tools are relatively minor. These statistical test results underscore the advantage of our approach in the comparative analysis. Results of the McNemar’s test have also been referenced in the “Performance on the RefSeq test set” section and detailed in Supplementary Section S8.

7. Language models have been used in previous bioinformatics studies i.e., PMID: 37381841, PMID: 38636332. Therefore, the authors are suggested to refer to more works in this description to attract a broader readership.
We thank the reviewer for this comment. We have incorporated references to various applications of language models within the field of bioinformatics in the Introduction section. The revised paragraph now reads as follows:
"Rapidly evolving language models have demonstrated significant advantages across various bioinformatics domains [13], including gene expression prediction [14], drug discovery [15], and tumor T-cell antigen (TTCA) classification [16]. Hence, as a major component of our methodology, we aim to leverage the language models to explore the modular structure of plasmids.”
We wish that introducing the applications of language models in bioinformatics would attract a wider readership.

8. The study should discuss any ethical considerations related to the use of public datasets, particularly regarding data privacy and consent if any sensitive data is involved. Furthermore, the potential commercial implications of the PlasGO tool, especially if it is used for proprietary research, should be addressed.
We thank the reviewer for pointing this out.
First, the primary dataset we utilized is the publicly available NCBI RefSeq plasmid database, which can be directly downloaded from the FTP site link (ftp.ncbi.nlm.nih.gov/genomes/refseq/plasmid/). The data included in this database is not associated with personal information or sensitive data. Therefore, there are no direct ethical considerations in the context of our work, and all the data we used complies with the terms and conditions set forth by NCBI for public use.
Second, the user-friendly PlasGO tool, along with the related data and code used in our experiments, is freely available on GitHub (https://github.com/Orin-beep/PlasGO) and the Zenodo repository (http://zenodo.org/records/14005015). As the manuscript was submitted to GigaScience, an open-access journal, the tool is intended for broad, unrestricted use. Thus, we do not foresee any direct commercial

implications arising from the use of PlasGO, as our focus remains on contributing to the public domain and supporting open science initiatives.
We have also addressed the above in a distinct section titled “Ethical considerations and potential commercial implications”.

9. While the manuscript mentions that PlasGO's code will be made available, it is crucial to ensure that all aspects of the research are fully reproducible.
We appreciate the reviewer for this comment. All codes and relevant data have been uploaded to the Zenodo repository (http://zenodo.org/records/14005015). This encompasses all experimental data and codes utilized in the PlasGO paper, along with the scripts for preprocessing datasets, training models, and the user-friendly and end-to-end PlasGO tool implemented with Python.

10. The hierarchical architecture and the use of extensive training data might lead to overfitting, especially given the high dimensionality of the input features. The manuscript should discuss the measures taken to prevent overfitting, such as regularization techniques, dropout, or cross-validation strategies.
We appreciate the reviewer for this comment. The training of the PlasGO models involved the utilization of various methods, outlined as follows:
1) Dropout: dropout layers were applied after the token embedding layers, the multi-head self-attention layers, the feed-forward network within the Transformer encoders, and the contextualized embeddings learned by the BERT module;
2) Model simplification: While BERT was utilized to capture plasmid modular patterns, we opted for its hyperparameters aligned with the size of the GO term dataset. For instance, we employed 4 Transformer encoder layers for the Molecular Function (MF) and Biological Process (BP) categories and 2 layers for the Cellular Component (CC) category. This decision was based on the relatively smaller dataset and label size for the CC category;
3) Regularization technique: a rank regularization (RR) loss is integrated into the total loss function. This regularization method, elaborated in Supplementary Section S1, aimed to enhance the model's capacity to differentiate between low-confidence and high-confidence predictions. Additionally, the model was prevented from overfitting by discouraging the assignment of high confidence scores to all predictions;
4) Early stopping: the training process will stop if the performance on the validation begins to deteriorate;
5) Cross-validation: a 5-fold cross-validation benchmark experiment was carried out, as outlined in Supplementary Section S10. The performance comparison is depicted in Supplementary Figure S5.
The five methods employed to prevent overfitting have been mentioned in the “PlasGO model training” section and summarized in Supplementary Section S3.

11. The study could benefit from a more detailed discussion on the practical implications of using PlasGO in real-world plasmid research. How can this tool be integrated into existing workflows for plasmid function prediction? What are the potential limitations in practical applications?
We thank the reviewer for this suggestion. PlasGO is implemented as an end-to-end and user-friendly tool available on GitHub at https://github.com/Orin-beep/PlasGO. It can accept both complete plasmids and plasmid-borne contigs as inputs, generating high-confidence GO annotations for the proteins they encode. Users have the flexibility to provide meticulously curated plasmids from public databases like PLSDB [17] and IMG/PR [18]. Conversely, they can also initiate the analysis from diverse data sources, spanning isolates, metagenomes, metatranscriptomes, and single amplified genomes. Many tools, such as PLASMe [19] and MOB-recon [20], support the identification of plasmid-borne contigs from sequencing data. PlasGO can then integrate with gene recognition tools like Prodigal [21] to annotate protein functions for the input contigs.
The predicted function annotations for plasmid-encoded proteins play a pivotal role in a range of downstream plasmid-related tasks. These tasks include plasmid mobility classification, plasmid replication mechanism prediction, and identifying accessory traits such as antibiotic resistance, virulence, and resistance to heavy metals. Furthermore, PlasGO can offer insights into more intricate aspects of plasmid research, encompassing host prediction and tracing the pathways of plasmid exchange among bacteria.
In practical applications, a potential limitation of PlasGO is the existence of elusive labels, which refer to a few GO terms that are difficult to predict accurately, as detailed in the “Identification of elusive GO term labels” section within the Results part. This limitation mainly stems from the scarcity of training samples, potentially impacting PlasGO's performance in two ways. First, the constrained diversity in the training data for these elusive labels presents challenges for the model in effectively capturing their distinct features,

thus hindering its ability to generalize to unseen proteins. Second, the imbalance between positive (proteins with the GO term) and negative samples (proteins without the GO term) associated with the elusive labels may lead to the model overfitting on negative samples and underfitting on positive samples. As a result, the model tends to predict elusive labels in a cautious manner, often refraining from positive predictions unless entirely confident.
To tackle this limitation, we have implemented two strategies. Firstly, we incorporated two usage modes for the PlasGO tool: the alignment-based mode and the learning-based mode. In the alignment-based mode, users can leverage Diamond to search against our curated high-confidence protein database using a strict E-value cutoff (e.g., 1e-5). Although we have filtered out "elusive" PlasGO predictions, the original high-quality RefSeq annotations for the elusive labels are retained. Consequently, the alignment offers users precise annotations for these elusive labels when their query proteins exhibit homology to the corresponding target proteins. Secondly, the majority of the elusive labels have their ancestor terms reversed (not designated as "elusive"), and the nearest ancestor terms of most elusive labels maintain good performance in terms of the AUPR metric. Thus, PlasGO can effectively predict the functions of elusive labels by prioritizing a broader representation of gene product attributes, albeit sacrificing some resolution. Looking ahead, as high-quality GO annotations continue to expand rapidly, we anticipate that the PlasGO model will greatly benefit from the augmented training samples. This enhancement is expected to boost the performance of elusive labels, ultimately transforming them from "elusive" to labels that can be predicted with confidence. The training scripts for PlasGO have been made available to users and readers, enabling them to customize their own PlasGO models. Furthermore, we commit to regularly updating our model version by integrating the expanding protein data. All the above analyses have been incorporated at the end of the Discussion section.

Reviewer 2:
In this paper, the authors introduce "PlasGO," a language model for GO annotation of plasmid proteins. The model takes into account two levels of representation: (1) the amino acid level, producing embeddings of the analyzed proteins based on a foundation protein language model, and (2) the plasmid gene level, where the aa-based embeddings are considered as part of a language model representing each protein in the genetic context in which it is encoded. This approach leverages the modular organization of different functions on plasmid genomes. Benchmarking performed by the authors against other deep-learning GO annotation algorithms demonstrates a considerable improvement of PlasGO over existing methods. The research is timely, well-performed, and clearly explained.
We thank the reviewer for the positive feedback and are delighted to know that our work is helpful.

- Major comments
1. The authors acknowledge that only a relatively small portion of the proteins in their database have GO term annotations, which may limit the model's ability to learn plasmid patterns effectively. As they correctly point out, an iterative approach could be useful to improve performance. Specifically, high-confidence GO annotations predicted by PlasGO could be used as input for another round of prediction, and this process can be repeated until no new reliable predictions are produced. Given that the authors have all the data and models required to run such an iterative search, I would warmly recommend doing so and reporting if and how the predictions improve.
We are grateful to the reviewer for this valuable suggestion, and we recognize the benefits of an iterative approach. An example of this is the popular iterative protein search tool PSI-BLAST [22], which enhances sensitivity in identifying distant protein homologs by iteratively refining its database (position-specific scoring matrix) with new sequences discovered in each iteration.
However, the iterative approach akin to PSI-BLAST cannot be directly applied to the PlasGO model. As demonstrated in Figure 2 of the main text, during both training and prediction phases, the PlasGO model receives the same input—protein embeddings arranged in the order of their encoding in the plasmid. Notably, although the unannotated proteins' predicted probabilities do not contribute to the loss calculation due to the lack of GO annotation labels, their embedding remains a crucial input for the model. Consequently, if the parameters of the PlasGO model remain unchanged, the predicted GO probabilities for proteins will also remain constant.
To incorporate the concept of iterative search into our model, we designed an iterative fine-tuning strategy with high-confidence pseudo-labeling. The pseudocode for this strategy is detailed in Supplementary Algorithm S1. The PlasGO model undergoes initial training with 10 epochs on the original training set, aiming to produce high-quality predictions essential for serving as pseudo-labels in subsequent fine-tuning

stages. Rather than employing a static threshold for selecting high-confidence pseudo-labels, the iterative fine-tuning process enables the model to dynamically learn the optimal confidence cutoff for improved prediction accuracy. Specifically, prior to the first round of fine-tuning, we employ the initialized model to predict GO probabilities and their associated confidence scores for all unannotated proteins. Then, the top 10% (iteration number divided by 10) of the most confident predicted GO probabilities are selected as soft pseudo-labels, while the remaining 90% of unannotated labels are still masked during loss calculation. These selected soft pseudo-labels are integrated with the original training labels, and the model is fine-tuned on this augmented dataset with a lower learning rate of 5e-5 for a single epoch. In the subsequent round, the fine-tuned model from the previous iteration is used to generate pseudo-labels, with a gradual relaxation of the confidence threshold (e.g., 20% for the second round). Early stopping is employed based on the model's performance on the validation set. If there is a performance decrease in any round or the maximum of 10 rounds is reached, the iterative fine-tuning process concludes.

We assess the performance of PlasGO with the proposed iterative fine-tuning strategy against initial training alone on the RefSeq test set. The results in Supplementary Table S3 reveal that the iterative fine-tuning approach demonstrated a modest improvement across all GO categories and evaluation metrics, with the most notable enhancement of 1.8% observed in the Fmax metric within the Molecular Function (MF) category. Moreover, early stopping is activated at the sixth round for the MF and Biological Process (BP) categories. This observation indicates that the top 60% of predictions exhibit sufficient confidence and quality to be utilized as pseudo-labels for these two categories. In summary, these findings display the effectiveness of leveraging augmented datasets with pseudo-labels in enhancing GO prediction accuracy through learning plasmid patterns.

To delve deeper into the reasons behind the lack of significant improvement (>2%) with the iterative fine-tuning strategy, one potential explanation could be that PlasGO has already predicted accurately for a substantial portion of samples. Consequently, the remaining misclassified samples may represent the more challenging cases. We choose Supplementary Figure S1 (a sub-figure from Supplementary Figure S6) for illustration. In this depiction, the contextualized embeddings learned by PlasGO reveal a scenario where certain positive samples (depicted by blue dots, corresponding to proteins annotated with "response to antibiotics") are intermingled with negative samples (represented by red dots, correlating to proteins without the "response to antibiotics" annotation). Despite efforts to augment our datasets with high-confidence pseudo-labels, it's still difficult to learn a clear boundary for explicitly classifying these intricate cases.

We believe in the significance of integrating the iterative search concept. Consequently, we have incorporated the iterative fine-tuning method as a post-training phase within the "PlasGO model training" section, and elaborated upon in Supplementary Section S5. Additionally, we have made the code "train_plasgo_iterative.py" and the models trained with this strategy available to our users and readership at the Zenodo repository (http://zenodo.org/records/14005015).

2. The gLM model (Hwang et al.) is highly similar to PlasGO in terms of the general approach, combining protein embedding (ESM2 in gLM) with genomic contextual data. Discussing the differences between the approaches and comparing their performances would provide important context and highlight the novelty of PlasGO.

We appreciate the reviewer for this suggestion. gLM is a pre-trained genomic language model, which integrates ESM and RoBERTa, and undergoes training on millions of metagenomic scaffolds. While gLM and PlasGO have a similar model architecture, namely a BERT family model built upon protein language models (PLMs), their design concepts and training methodologies are different. gLM functions as a transfer-learning-based foundational model designed at the genomic level (distinct from PLMs that operate at the protein level), capable of generating protein-level and contig-level embeddings for various downstream tasks [1]. The authors of gLM conducted an experiment involving the prediction of Enzyme Commission (EC) numbers for proteins in their paper [1], a task closely related to our GO term prediction objective. Specifically, a linear probe (LP) is trained using the embeddings derived from gLM on the EC-labeled dataset. Hence, we apply the same methodology used for EC number prediction in gLM's paper to our GO term prediction tasks for comparison with PlasGO, followed by an analysis of the distinctions between the two tools.

We assessed gLM's performance on plasmid-encoded proteins through two approaches. First, we generated protein-level embeddings by feeding gLM with plasmid segments containing up to 30 proteins. Then, we trained a linear probe using gLM's contextualized embeddings on the identical dataset with PlasGO. The performance evaluation comparing PlasGO and gLM is detailed in Supplementary Table S4. Second, since

gLM also functions as a foundation model, we trained an additional version of PlasGO utilizing gLM's contextualized embeddings as input for PlasGO's core BERT module. This benchmark follows the methodology (with the initial 10-epoch training only) outlined in the section "Ablation studies: validating PlasGO's design rationale" in the PlasGO paper. The performance comparison between PlasGO trained with ProtT5 (standard) and gLM is presented in Supplementary Table S5.
As shown in Supplementary Table S4, we can observe that the contextualized embeddings generated by gLM exhibit a weak correlation with GO annotations for plasmid-encoded proteins. This suggests that knowledge gained from the general metagenomic corpus by gLM (across all biological entities) offer limited enhancements for plasmid-specific protein tasks. Furthermore, it proves the significance of designing PlasGO, a tool specialized for protein annotation within the plasmid domain. As for the second experiment (Supplementary Table S5), while PlasGO can enhance predictions when combined with gLM as opposed to solely training a linear probe, the performance is not yet optimal. Therefore, integrating PlasGO's BERT module for capturing plasmid modular patterns with a robust PLM such as ProtTrans or ESM remains the most effective strategy.
For a more in-depth discussion, gLM's design is reasonably grounded, as a rich dataset (7.32 million sub-contigs covering a wide range of taxonomies and biological entities) can be used for its masked language modeling pretraining. However, plasmid-borne contigs constitute a very small portion of gLM's pretraining corpus, which may result in underfitting and hinder the model's ability to learn plasmid-specific genomic context information. On the other hand, although we represent plasmids as a language defined by proteins, the syntax of plasmids might differ significantly from that of other biological entities due to the specialized mechanisms of plasmids (e.g., conjugative transfer). Consequently, the knowledge gained from the general metagenomic corpus by gLM may have limited impact on improving the prediction of functions for plasmid-encoded proteins.
Another difference lies in the training methodologies employed by gLM and PlasGO. In the process of training a linear probe for function prediction, each protein is individually trained without incorporating contextualized information, potentially leading to the loss of genomic context knowledge gained during pretraining. In contrast, PlasGO consistently undergoes training on the GO-labeled plasmid corpus, facilitating a more thorough learning and retention of contextual information to enhance functional predictions. Finally, given the complexity of GO term prediction involving a vast number of GO labels in a multi-label classification setting, we enhance PlasGO by incorporating a self-attention confidence weighting mechanism, enabling the evaluation of a confidence score for each GO prediction. As demonstrated in our paper, these confidence scores play a crucial role in generating reliable GO predictions for users. The above experiments and discussions comparing PlasGO and gLM are also elaborated in Supplementary Section S7.

3. The agreement of the PlasGO prediction with the GO terms retrieved from sequence databases ("ground truth") was determined by calculating the ratio of terms shared between the high-confidence predictions and ground truth, divided by the number of high-confidence predictions. This measure is asymmetrical and might generate over-optimistic results. At the extreme, if the algorithm produces a very large number of predictions, this value will tend to be very high just because there are many more GO terms predicted than GO terms in the ground truth. I strongly recommend using a symmetrical measure, such as the Jaccard index.
We thank the reviewer for this thoughtful suggestion, and we recognize that the symmetrical metric can provide a clearer reflection of PlasGO's predictive accuracy. As a result, we have substituted the original asymmetrical metric with the Jaccard index in the case study experiment. Specifically, for each test protein, we now calculate a Jaccard index between the high-confidence GO term set predicted by PlasGO and the potential ground truth set obtained from the Swiss-Prot database. The updated results are presented in Figure 11 of the main text.
As expected, the symmetrical Jaccard index values exhibit a slightly lower overall level and greater fluctuation compared to the results evaluated using the original asymmetrical precision metric, which could be over-optimistic. However, among the 76 test proteins, 62 (~81.6%) have a Jaccard index exceeding 0.7, indicating a high resemblance between the predicted high-confidence GO terms and the retrieved ground truth set. This proves PlasGO's capability to provide reliable GO annotations for the two complete plasmid case studies.

4. The methodology for calculating average precision and recall is potentially skewed. The authors compute average precision over proteins with at least one annotation, ignoring proteins lacking annotation (instead of counting these as misclassifications). This approach makes sense given that numerous plasmid proteins

lack GO annotations. However, the average recall is calculated across all proteins (N). For unannotated proteins, the correct classification is not defined. Since these cases are also considered in the measure of recall, I assume PlasGO high-confidence predictions were considered correct. This seems like a problematic assumption that might lead to skewed results. I would therefore suggest that unannotated proteins be omitted from the recall calculation, as was done in the precision calculation.
We thank the reviewer for the question. Based on the common practice of computing Fmax, the unannotated proteins were all excluded from the Fmax metric calculation, given the absence of ground truth for evaluating prediction performance on these proteins. In line with the CAFA3 challenge [4], a recognized benchmark for validating computational tools in protein function prediction, we adhered to the standard definition of Fmax. The original formula for this metric can be referenced in “Additional file 1” of the CAFA paper [4].
To be specific, Fmax is computed based on the probability vectors predicted by a tool and the ground-truth label encoding vectors containing binary values (0s and 1s). It is determined as the maximum F1-score achieved using arbitrary cutoffs applied to the probability vectors, varying between 0 and 1. Hence, in Equation 6 of the main text, $m(\theta)$ represents the count of proteins with at least one label’s predicted probability exceeding the cutoff θ, rather than the proteins with at least one annotation. For example, at a cutoff θ of 0, $m(\theta)$ equals n (the total number of test proteins). Conversely, at a very high cutoff value θ near 1, $m(\theta)$ will be lower.
For obtaining Fmax, we compute the F1-score for each θ value within the range of 0 to 1, with an increment of 0.01, and identify the highest score as Fmax. Empirically, the Fmax metric reaches its peak when the cutoff θ is approximately 0.3, leading many tools to adopt 0.3 as the default probability threshold for GO term prediction. To exemplify, we utilize PlasGO’s predicted probability vector within the Molecular Function (MF) category on the RefSeq test set. The variations in average precision and recall, F1-score, and the $m(\theta)$ values are delineated in Supplementary Figure S2 across the spectrum of the cutoff θ from 0 to 1. We can observe that as the cutoff θ rises, the average precision increases, while the average recall and $m(\theta)$ decline. The F1-score peaks at 0.8250 when θ equals 0.26. These findings are consistent with the above discussion. The above discussion regarding the Fmax metric has been included in Supplementary Section S6.

5. The authors identify and filter out "elusive" GO terms that are difficult to predict. This is reasonable in the scope of this paper, but since it is still a central limitation of PlasGO, I would suggest discussing (even if not implementing) approaches to improve the predictions in these challenging cases.
We thank the reviewer for this comment. The presence of elusive labels can primarily be attributed to the scarcity of training samples, potentially hindering PlasGO’s performance in two ways. Firstly, the limited diversity within the training data for these elusive labels poses challenges for the model to effectively capture their distinctive features, thus impeding its ability to generalize to unseen proteins. Secondly, the imbalance between positive (proteins with the GO term) and negative samples (proteins without the GO term) associated with elusive labels may result in the model overfitting on negative samples and underfitting on positive samples. Consequently, the model tends to predict elusive labels in a “conservative” fashion, often refraining from positive predictions unless entirely confident.
To address this issue, we have implemented two strategies. Firstly, we introduced two usage modes for the PlasGO tool: the alignment-based mode and the learning-based mode. In the alignment-based mode, users can leverage Diamond to conduct searches against our curated high-confidence protein database using a strict E-value cutoff (e.g., 1e-5). Notably, within our curated database, while we have filtered out the “elusive” PlasGO predictions, the original high-quality RefSeq annotations for the elusive labels are reserved. As a result, the alignment offers users precise annotations for these elusive labels when their query proteins exhibit homology to the corresponding target proteins. Secondly, the majority of the elusive labels have their ancestor terms reversed (not designated as “elusive”). As shown in Supplementary Figure S11, the nearest ancestor terms of most elusive labels maintain good performance in terms of the AUPR metric. Thus, PlasGO can effectively predict the functions of elusive labels by prioritizing a broader representation of gene product attributes, albeit sacrificing some resolution.
Looking ahead, as high-quality GO annotations continue to expand rapidly, we anticipate that the PlasGO model will greatly benefit from the augmented training samples. This enhancement is expected to boost the performance of elusive labels, ultimately transforming them from “elusive” to labels that can be predicted with confidence. The training scripts for PlasGO have been made available to users and readers, enabling them to customize their own PlasGO models. Furthermore, we commit to regularly updating our model version by integrating the expanding protein data.

We have revised the discussion in the “Identification of elusive GO term labels” section accordingly.

6. In Figures 8 and 9, a perfect AUPR of 1 is reported in several cases. Such perfect classification performances are highly unusual and warrant an examination to double-check this result and if it persists discuss the underlying reasons for these perfect results.
We thank the reviewer for this comment. As shown in Figure 8 of the main text, the AUPR performance for each Molecular Function (MF) label is depicted in an order organized by their frequency of occurrence in the training set, owing to the imbalance within the GO term dataset. Consequently, labels linked to a smaller number of training proteins also tend to have fewer corresponding test proteins. For instance, the top 10 MF labels (ranging from “MF1” to “MF10”) average 4,544 associated test proteins out of a total of 17,369 test proteins. Conversely, the five MF labels at the tail of Figure 8, each achieving a perfect AUPR of 1, are as follows: “GO:1901505” (carbohydrate derivative transmembrane transporter activity), “GO:0033218” (amide binding), “GO:0097351” (toxin sequestering activity), “GO:0140313” (molecular sequestering activity), and “GO:0140911” (pore-forming activity). These labels have an average of 18 test proteins. As a result, the AUPR values for high-frequency labels at the start of Figure 8 are more stable, whereas those for low-frequency labels are more susceptible to random chance and tend to exhibit significant fluctuations. This explains why certain low-frequency labels can achieve perfect performance.

7. The masking approach during training is not entirely clear. If I understand correctly, annotated proteins are being masked during prediction. This is expected to lead to the loss of a lot of contextual information. On the other hand, during training, the unannotated proteins are masked, losing potentially informative sequence data. I would suggest splitting complete plasmids between train/test/validation sets, and if needed, performing cross-validation to cover the entire dataset. This way for each plasmid the entire protein sequence and context information will be used.
Thanks for this comment. We acknowledge that the inclusion of gray arrows in the figure illustrating PlasGO’s workflow could potentially be misleading. Consequently, we have updated the figure by removing the gray arrows, and the revised version is presented in Figure 2 in the main text.
Masking is solely applied to proteins that are not present in the training set when calculating the loss function during training, given their lack of labels hindering their involvement in backpropagation. Conversely, throughout both training and prediction, embeddings for all proteins encoded on a plasmid remain unmasked and are input into the PlasGO model, ensuring no loss of informative genomic context during each phase. We opted to split the dataset in terms of proteins rather than plasmids to simulate the real-world scenarios where plasmid sequence data is available while a majority of the encoded proteins lack annotations. Consequently, PlasGO can accept the same plasmid as input for both supervised training and prediction, focusing on different proteins in each phase—computing losses using proteins with GO labels and generating GO predictions for those lacking labels.
Nevertheless, employing plasmid-based data splitting and cross-validation also represent viable and beneficial approaches for showcasing the performance of PlasGO. Thus, we conducted a 5-fold cross-validation for PlasGO and the top three benchmarked tools. Firstly, we randomly divided all complete plasmids into five equal partitions, with each partition assigned to a fold. Within each fold, the annotated proteins from the designated partition comprised the test set, while the remaining annotated proteins constituted the training and validation sets. Aligning with the rule detailed in the “Data curation and model training” section regarding novel protein benchmark experiments, we ensured no significant alignment between the training and test sets. The performance, evaluated using Fmax and AUPR metrics, was averaged across the five groups, and the benchmarking results are illustrated in Supplementary Figure S5. We can observe that the benchmark results averaged from the 5-fold cross-validation align closely with the results presented in the “Performance on the RefSeq test set” section, with PlasGO outperforming other tools in Fmax and AUPR across all three GO categories. Compared to the single dataset split strategy, the cross-validation can provide a more reliable evaluation of PlasGO’s capacity for generalization to novel proteins. This experiment has been included in Supplementary Section S10. Moreover, the discussions above are referenced in the “Design rationale”, “RefSeq dataset”, and “Performance on the RefSeq test set” sections.

8. There seems to be somewhat of a contradiction between the two following statements appearing in the paper: (1) "CaLM, despite being a pre-trained PLM, did not surpass the top three tools using ProtTrans, which is consistent with the results reported in CaLM's paper" and (2) "Experimental results demonstrate that the protein representations derived from CaLM outperform other PLMs in the classification of GO

terms." Furthermore, other PLMs, such as ESM, performed better at GO annotation prediction according to the CaLM paper. These might have been more appropriate for this task. CodonBERT, a codon-based PLM also based on ProtTrans, could also have been a suitable alternative.
We thank the reviewer for catching this, and we recognize the inconsistency between the descriptions in the Introduction and Results sections. Given that the ESM-2 family models exhibit superior performance, as demonstrated in the CaLM paper [23], we have substituted CaLM with ESM-2 as the example for PLMs in the Introduction, as stated below:
"As an example, the ESM-2 family models utilize a BERT-style encoder variant with up to 15 billion parameters, making them the largest PLMs to date. After training with an unsupervised masked language modeling (MLM) objective on a dataset comprising over 60 million protein sequences, ESM-2 demonstrates superior performance in the classification of GO terms."
Additionally, we included ESM-2 and CodonBERT for benchmarking to enhance the presentation of our results. In particular, we selected the model "esm2_t36_3B_UR50D" with 36 Transformer layers from the ESM-2 family models to maintain consistency with the ProtT5 model employed for PlasGO, both of which consist of 3 billion parameters. The benchmarking methods employed for them were aligned with the original CaLM. The updated comparative results are illustrated in Figure 6 of the main text.
PlasGO consistently demonstrated the best performance across all three GO categories, with PFresGO, ESM-2, and TM-Vec emerging as the subsequent top-performing tools, all leveraging PLMs. The results prove the pivotal role of PLMs in enhancing protein function prediction by capturing inherent relationships between amino acids. This aligns with the finding that many proteins execute their functions through spatially aggregated clusters of critical residues [24]. Furthermore, the performance of codon-based large language models, including both the original CaLM and CodonBERT, did not surpass the top four tools utilizing PLMs. This suggests that training on codon-level tokens does not offer the same enhancements as training on amino acid-level tokens for function prediction tasks. Nevertheless, this observation does not negate the superior advantages of codon-based models in specific tasks such as protein expression prediction or protein abundance prediction. The modifications made to the benchmarking experiments have been updated to the "Performance on the RefSeq test set" section.

- Minor issues
- To improve the reading flow of the paper, consider reordering the ablation section to precede the "Performance on the RefSeq test set" section, since the ablation studies section provides the rationale for the choices of architecture and foundation protein language model.
We thank the reviewer for this suggestion. We have relocated the ablation section before the "Performance on the RefSeq test set" section, enhancing the overall coherence of the paper.

- "We initially downloaded all available plasmids from the NCBI RefSeq database" - I would suggest specifying the query or approach used to acquire all plasmids from RefSeq.
We thank the reviewer for this comment. All available plasmid sequences, their encoded protein sequences, and the associated annotation files in both GBFF and GPFF formats can be directly downloaded from the NCBI RefSeq plasmid database FTP site at ftp.ncbi.nlm.nih.gov/genomes/refseq/plasmid/. Additionally, a bash script (download_refseq_plasmids.sh) for downloading the plasmid data, along with a Python script (preprocess_refseq_plasmids.py) for preprocessing the data—including the selection of high-quality plasmids— are both available in the Zenodo repository at http://zenodo.org/records/14005015 to facilitate further research. A description for querying the plasmids has also been added to the corresponding paragraph in the main text.

- I would recommend using the term "protein embedding" instead of "protein token," which may be misleading. The term "token embeddings" used in Figure 3 is more accurate than "protein token," and yet "protein embeddings" is probably the most accurate term in this case.
We thank the reviewer for pointing this out. We have replaced all instances of the term "protein token" with "protein embedding" in the main text, as well as Figure 2 and 3 in the main text.

- Figure 1: To provide an accurate depiction of representative plasmids, I suggest including unannotated genes in Figure 1.
We appreciate the reviewer's comment. We have included the unannotated genes in Figure 1, represented as pentagon blocks with no color. Since the majority of genes (42 out of 56, excluding pseudogenes) encoded on the well-characterized plasmid pOLA52 [25] have gene product annotations retrieved from

NCBI, this plasmid serves as a qualified example for illustrating the plasmid's modularization pattern to the readership.

- Figure 4: "Global average pooling" was misspelled.
We thank the reviewer for catching this error, and we have now corrected the typo in Figure 4.

- Figure 10: "The prediction precision of PlasGO is determined by calculating the ratio of the number of proteins in set A that are also present in set B to the total number of predicted high-confidence proteins (|A|)". If I understand the figure correctly, it should be "number of GO terms" instead of "number of proteins" in both cases.
We appreciate the reviewer for pointing this out, and we acknowledge that the term "number of GO terms" should be correct in its original context. However, as we have employed the symmetrical Jaccard index as our evaluation metric, we updated the corresponding sentence in the caption of Figure 10 (of the main text) as follows: "The predictive accuracy of PlasGO is evaluated by calculating the Jaccard index between the predicted high-confidence GO term set A and the potential ground truth set B."

- A figure (or supplementary figure) depicting one of the plasmids with some of the high-confidence predictions in the case study section (along the same lines as Figure 1 but with a distinction between previously known and unknown annotations) could enhance the clarity and impact of the results.
We appreciate the reviewer for the valuable suggestion. We have included a figure that illustrates the comparison between the GO annotations for plasmid pSK41 (one of the case studies), sourced from the raw RefSeq database and the predictions generated by PlasGO in Supplementary Section 20. As depicted in Supplementary Figure S15, the raw RefSeq database contains GO annotations for only two proteins within the payload functional class. In contrast, PlasGO effectively assigned GO annotations to all proteins encoded on plasmid pSK41, with the exception of two proteins (highlighted by purple pentagon blocks) that could not be categorized into the five functional classes using the GO term indicators. We believe that this approach will improve the clarity in showcasing our tool's performance in annotating plasmid-encoded proteins.

References

[1]. Hwang, Yunha, et al. "Genomic language model predicts protein co-regulation and function." Nature communications 15.1 (2024): 2880.
[2]. O'Leary, Nuala A., et al. "Reference sequence (RefSeq) database at NCBI: current status, taxonomic expansion, and functional annotation." Nucleic acids research 44.D1 (2016): D733-D745.
[3]. Jiang, Yuxiang, et al. "The impact of incomplete knowledge on the evaluation of protein function prediction: a structured-output learning perspective." Bioinformatics 30.17 (2014): i609-i616.
[4]. Zhou, Naihui, et al. "The CAFA challenge reports improved protein function prediction and new functional annotations for hundreds of genes through experimental screens." Genome biology 20 (2019): 1-23.
[5]. Meier, Joshua, et al. "Language models enable zero-shot prediction of the effects of mutations on protein function." Advances in neural information processing systems 34 (2021): 29287-29303.
[6]. Pan, Tong, et al. "PFresGO: an attention mechanism-based deep-learning approach for protein annotation by integrating gene ontology inter-relationships." Bioinformatics 39.3 (2023): btad094.
[7]. Dewan, Ian, and Hildegard Uecker. "A mathematician's guide to plasmids: an introduction to plasmid biology for modellers." Microbiology 169.7 (2023): 001362.
[8]. Carattoli, Alessandra, and Henrik Hasman. "PlasmidFinder and in silico pMLST: identification and typing of plasmid replicons in whole-genome sequencing (WGS)." Horizontal gene transfer: methods and protocols (2020): 285-294.
[9]. Thomas, Christopher M., et al. "Annotation of plasmid genes." Plasmid 91 (2017): 61-67.
[10]. Shintani, Masaki, and Haruo Suzuki. "Plasmids and their hosts." DNA Traffic in the Environment (2019): 109-133.
[11]. Shang, Jiayu, et al. "Accurate and efficient protein embedding using multi-teacher distillation learning." Bioinformatics 40.9 (2024): btae567.
[12]. Japkowicz, Nathalie, and Mohak Shah. Evaluating learning algorithms: a classification perspective. Cambridge University Press, 2011.
[13]. Le, Nguyen Quoc Khanh. "Leveraging transformers-based language models in proteome bioinformatics." Proteomics 23.23-24 (2023): 2300011.

[14]. Khan, Anwar, and Boreom Lee. "DeepGene transformer: Transformer for the gene expression-based classification of cancer subtypes." Expert Systems with Applications 226 (2023): 120047.
[15]. Huang, Kexin, et al. "MolTrans: molecular interaction transformer for drug–target interaction prediction." Bioinformatics 37.6 (2021): 830-836.
[16]. Tran, Thi-Oanh, and Nguyen Quoc Khanh Le. "Sa-ttca: an svm-based approach for tumor t-cell antigen classification using features extracted from biological sequencing and natural language processing." Computers in Biology and Medicine 174 (2024): 108408.
[17]. Schmartz, Georges P., et al. "PLSDB: advancing a comprehensive database of bacterial plasmids." Nucleic Acids Research 50.D1 (2022): D273-D278.
[18]. Camargo, Antonio Pedro, et al. "IMG/PR: a database of plasmids from genomes and metagenomes with rich annotations and metadata." Nucleic acids research 52.D1 (2024): D164-D173.
[19]. Tang, Xubo, et al. "PLASMe: a tool to identify PLASMid contigs from short-read assemblies using transformer." Nucleic Acids Research 51.15 (2023): e83-e83.
[20]. Robertson, James, and John HE Nash. "MOB-suite: software tools for clustering, reconstruction and typing of plasmids from draft assemblies." Microbial genomics 4.8 (2018): e000206.
[21]. Hyatt, Doug, et al. "Prodigal: prokaryotic gene recognition and translation initiation site identification." BMC bioinformatics 11 (2010): 1-11.
[22]. Altschul, Stephen F., et al. "Gapped BLAST and PSI-BLAST: a new generation of protein database search programs." Nucleic acids research 25.17 (1997): 3389-3402.
[23]. Outeiral, Carlos, and Charlotte M. Deane. "Codon language embeddings provide strong signals for use in protein engineering." Nature Machine Intelligence 6.2 (2024): 170-179.
[24]. Lichtarge, Olivier, Henry R. Bourne, and Fred E. Cohen. "An evolutionary trace method defines binding surfaces common to protein families." Journal of molecular biology 257.2 (1996): 342-358.
[25]. Norman, Anders, et al. "Nucleotide sequence of pOLA52: a conjugative IncX1 plasmid from Escherichia coli which enables biofilm formation and multidrug efflux." Plasmid 60.1 (2008): 59-74.
