# Supplementary material for: PlasGO: enhancing GO-based function prediction for plasmid-encoded proteins based on genetic structure
Source: Gigascience. 2024 Dec 20;13:giae104. doi: 10.1093/gigascience/giae104 (PMC11659980; doi:10.1093/gigascience/giae104)
Supplement: giae104_Response_to_Reviewer_Comments_Revision_1 [file giae104_response_to_reviewer_comments_revision_1.pdf]

Author's Response To Reviewer Comments

Dear Editor,

We would like to thank you for your thorough review of our paper and the valuable comments provided. We have addressed all of your suggestions. The revisions in the main draft are highlighted in red and can be summarized as follows:

1) We have included the URLs for our Zenodo dataset [1] and GigaDB [2] in the reference list and have cited them in the "Data Availability" section.
2) We have added the latest FTP link for the NCBI RefSeq plasmid database as a URL citation [3] in the reference list and cited it in the "Ethical Considerations and Potential Commercial Implications" section.
3) We have provided additional details in the "Funding" section, including the funding source, grant number, and the name of the Principal Investigator: "This work is supported by Research Grants Council (RGC), the University Grants Committee (UGC) of Hong Kong, General Research Fund (GRF) 11214924, Y Sun."
4) We have registered our PlasGO tool in the bio.tools database (identifier: "PlasGO") and the SciCrunch.org database (RRID: "SCR_025983"). This information has also been included in the "Availability of Source Code and Requirements" section.

References:

[1] Ji Y, PlasGO_dataset. Zenodo; 2024. https://doi.org/10.5281/zenodo.14005015.
[2] Ji Y, Shang J, Guan J, Zou W, Liao H, Tang X, et al., Supporting data for "PlasGO: enhancing GO-based function prediction for plasmid-encoded proteins based on genetic structure". GigaScience Database; 2024. http://gigadb.org/dataset/102621.
[3] Reference sequence (RefSeq) plasmid database at NCBI [Internet]. Bethesda (MD): National Library of Medicine (US), National Center for Biotechnology Information; 2004 – [cited 2024 Nov 25]. Available from: https://ftp.ncbi.nlm.nih.gov/genomes/refseq/plasmid/.
