# Supplementary material for: PlasGO: enhancing GO-based function prediction for plasmid-encoded proteins based on genetic structure
Source: Gigascience. 2024 Dec 20;13:giae104. doi: 10.1093/gigascience/giae104 (PMC11659980; doi:10.1093/gigascience/giae104)
Supplement: giae104_Reviewer_1_Report_Original_Submission — Nguyen Quoc Khanh Le -- 8/18/2024 Reviewed [file giae104_reviewer_1_report_original_submission.pdf]

Reviewer Report

Title: PlasGO: enhancing GO-based function prediction for plasmid-encoded proteins based on genetic structure

Version: Original Submission **Date:** 8/18/2024

Reviewer name: Nguyen Quoc Khanh Le

Reviewer Comments to Author:

1. The manuscript introduces PlasGO, which leverages a hierarchical architecture for GO term prediction in plasmid-encoded proteins. However, the novelty of the approach could be questioned. While the combination of protein language models and BERT for GO prediction is innovative, similar methods have been applied in other contexts.2. The study heavily relies on data from the RefSeq database, yet there is limited discussion on the quality and completeness of this data. The manuscript should address potential biases introduced by incomplete or incorrect GO annotations in the database. Moreover, the study uses protein sequences of up to 1K length, which might exclude relevant longer sequences, potentially limiting the model's applicability to all plasmid-encoded proteins.3. The manuscript claims that PlasGO can generalize well to novel proteins, but this claim is based on a specific dataset. The model's generalizability should be tested on more diverse and independent datasets, including plasmids from different bacterial species or environmental contexts.4. While the model's performance is quantitatively evaluated, the interpretability of the results remains unclear. The study should include an analysis of how well the model's predictions align with known biological functions and pathways. Additionally, it would be helpful to include examples where PlasGO provides novel insights that other models do not, thereby demonstrating its practical utility.5. The manuscript does not provide detailed information on the computational resources required to train and run PlasGO. Given the complexity of the model, there are potential concerns about its scalability, particularly for larger plasmid datasets or in settings with limited computational power.6. The manuscript compares PlasGO with several state-of-the-art tools, but the comparison might not be fully exhaustive. Additionally, statistical significance tests for performance differences should be provided to support the comparative analysis.7. Language models have been used in previous bioinformatics studies i.e., PMID: 37381841, PMID: 38636332. Therefore, the authors are suggested to refer to more works in this description to attract a broader readership.8. The study should discuss any ethical considerations related to the use of public datasets, particularly regarding data privacy and consent if any sensitive data is involved. Furthermore, the potential commercial implications of the PlasGO tool, especially if it is used for proprietary research, should be addressed.9. While the manuscript mentions that PlasGO's code will be made available, it is crucial to ensure that all aspects of the research are fully reproducible.10. The hierarchical architecture and the use of extensive training data might lead to overfitting, especially given the high dimensionality of the input features. The manuscript should discuss the measures taken to prevent overfitting, such as regularization techniques, dropout, or cross-validation strategies.11. The study could benefit from a more detailed discussion on the practical implications of using PlasGO in real-world plasmid research.

How can this tool be integrated into existing workflows for plasmid function prediction? What are the potential limitations in practical applications?

Level of Interest

Please indicate how interesting you found the manuscript: Choose an item.

Quality of Written English

Please indicate the quality of language in the manuscript: Choose an item.

Declaration of Competing Interests

Please complete a declaration of competing interests, considering the following questions:

- Have you in the past five years received reimbursements, fees, funding, or salary from an organisation that may in any way gain or lose financially from the publication of this manuscript, either now or in the future?
- Do you hold any stocks or shares in an organisation that may in any way gain or lose financially from the publication of this manuscript, either now or in the future?
- Do you hold or are you currently applying for any patents relating to the content of the manuscript?
- Have you received reimbursements, fees, funding, or salary from an organization that holds or has applied for patents relating to the content of the manuscript?
- Do you have any other financial competing interests?
- Do you have any non-financial competing interests in relation to this paper?

If you can answer no to all of the above, write 'I declare that I have no competing interests' below. If your reply is yes to any, please give details below.

None

I agree to the open peer review policy of the journal. I understand that my name will be included on my report to the authors and, if the manuscript is accepted for publication, my named report including any attachments I upload will be posted on the website along with the authors' responses. I agree for my report to be made available under an Open Access Creative Commons CC-BY license (http://creativecommons.org/licenses/by/4.0/). I understand that any comments which I do not wish to be included in my named report can be included as confidential comments to the editors, which will not be published.

Choose an item.

To further support our reviewers, we have joined with Publons, where you can gain additional credit to further highlight your hard work (see: https://publons.com/journal/530/gigascience). On publication of this paper, your review will be automatically added to Publons, you can then choose whether or not to claim your Publons credit. I understand this statement.

Yes Choose an item.
