# Supplementary material for: PlasGO: enhancing GO-based function prediction for plasmid-encoded proteins based on genetic structure
Source: Gigascience. 2024 Dec 20;13:giae104. doi: 10.1093/gigascience/giae104 (PMC11659980; doi:10.1093/gigascience/giae104)
Supplement: giae104_Reviewer_2_Report_Original_Submission — David Burstein -- 9/16/2024 Reviewed [file giae104_reviewer_2_report_original_submission.pdf]

Reviewer Report

Title: PlasGO: enhancing GO-based function prediction for plasmid-encoded proteins based on genetic structure

Version: Original Submission **Date:** 9/16/2024

Reviewer name: David Burstein

Reviewer Comments to Author:

In this paper, the authors introduce "PlasGO," a language model for GO annotation of plasmid proteins. The model takes into account two levels of representation: (1) the amino acid level, producing embeddings of the analyzed proteins based on a foundation protein language model, and (2) the plasmid gene level, where the aa-based embeddings are considered as part of a language model representing each protein in the genetic context in which it is encoded. This approach leverages the modular organization of different functions on plasmid genomes. Benchmarking performed by the authors against other deep-learning GO annotation algorithms demonstrates a considerable improvement of PlasGO over existing methods. The research is timely, well-performed, and clearly explained.Main issues:1. The authors acknowledge that only a relatively small portion of the proteins in their database have GO term annotations, which may limit the model's ability to learn plasmid patterns effectively. As they correctly point out, an iterative approach could be useful to improve performance. Specifically, high-confidence GO annotations predicted by PlasGO could be used as input for another round of prediction, and this process can be repeated until no new reliable predictions are produced. Given that the authors have all the data and models required to run such an iterative search, I would warmly recommend doing so and reporting if and how the predictions improve.2. The gLM model (Hwang et al.) is highly similar to PlasGO in terms of the general approach, combining protein embedding (ESM2 in gLM) with genomic contextual data. Discussing the differences between the approaches and comparing their performances would provide important context and highlight the novelty of PlasGO.3. The agreement of the PlasGO prediction with the GO terms retrieved from sequence databases ("ground truth") was determined by calculating the ratio of terms shared between the high-confidence predictions and ground truth, divided by the number of high-confidence predictions. This measure is asymmetrical and might generate over-optimistic results. At the extreme, if the algorithm produces a very large number of predictions, this value will tend to be very high just because there are many more GO terms predicted than GO terms in the ground truth. I strongly recommend using a symmetrical measure, such as the Jaccard index.4. The methodology for calculating average precision and recall is potentially skewed. The authors compute average precision over proteins with at least one annotation, ignoring proteins lacking annotation (instead of counting these as misclassifications). This approach makes sense given that numerous plasmid proteins lack GO annotations. However, the average recall is calculated across all proteins (N). For unannotated proteins, the correct classification is not defined. Since these cases are also considered in the measure of recall, I assume PlasGO high-confidence predictions were considered correct. This seems like a problematic assumption that might lead to skewed results. I would therefore suggest that unannotated proteins be

omitted from the recall calculation, as was done in the precision calculation.5. The authors identify and filter out "elusive" GO terms that are difficult to predict. This is reasonable in the scope of this paper, but since it is still a central limitation of PlasGO, I would suggest discussing (even if not implementing) approaches to improve the predictions in these challenging cases.6. In Figures 8 and 9, a perfect AUPR of 1 is reported in several cases. Such perfect classification performances are highly unusual and warrant an examination to double-check this result and if it persists discuss the underlying reasons for these perfect results.7. The masking approach during training is not entirely clear. If I understand correctly, annotated proteins are being masked during prediction. This is expected to lead to the loss of a lot of contextual information. On the other hand, during training, the unannotated proteins are masked, losing potentially informative sequence data. I would suggest splitting complete plasmids between train/test/validation sets, and if needed, performing cross-validation to cover the entire dataset. This way for each plasmid the entire protein sequence and context information will be used.8. There seems to be somewhat of a contradiction between the two following statements appearing in the paper: (1) "CaLM, despite being a pre-trained PLM, did not surpass the top three tools using ProtTrans, which is consistent with the results reported in CaLM's paper" and (2) "Experimental results demonstrate that the protein representations derived from CaLM outperform other PLMs in the classification of GO terms." Furthermore, other PLMs, such as ESM, performed better at GO annotation prediction according to the CaLM paper. These might have been more appropriate for this task. CodonBERT, a codon-based PLM also based on ProtTrans, could also have been a suitable alternative.Minor issues:- To improve the reading flow of the paper, consider reordering the ablation section to precede the "Performance on the RefSeq test set" section, since the ablation studies section provides the rationale for the choices of architecture and foundation protein language model.- "We initially downloaded all available plasmids from the NCBI RefSeq database" - I would suggest specifying the query or approach used to acquire all plasmids from RefSeq.- I would recommend using the term "protein embedding" instead of "protein token," which may be misleading. The term "token embeddings" used in Figure 3 is more accurate than "protein token," and yet "protein embeddings" is probably the most accurate term in this case.- Figure 1: To provide an accurate depiction of representative plasmids, I suggest including unannotated genes in Figure 1.- Figure 4: "Global average pooling" was misspelled.- Figure 10: "The prediction precision of PlasGO is determined by calculating the ratio of the number of proteins in set A that are also present in set B to the total number of predicted high-confidence proteins (|A|)". If I understand the figure correctly, it should be "number of GO terms" instead of "number of proteins" in both cases.- A figure (or supplementary figure) depicting one of the plasmids with some of the high-confidence predictions in the case study section (along the same lines as Figure 1 but with a distinction between previously known and unknown annotations) could enhance the clarity and impact of the results.

Level of Interest

Please indicate how interesting you found the manuscript: Choose an item.

Quality of Written English

Please indicate the quality of language in the manuscript: Choose an item.

Declaration of Competing Interests

Please complete a declaration of competing interests, considering the following questions:

- Have you in the past five years received reimbursements, fees, funding, or salary from an organisation that may in any way gain or lose financially from the publication of this manuscript, either now or in the future?
- Do you hold any stocks or shares in an organisation that may in any way gain or lose financially from the publication of this manuscript, either now or in the future?
- Do you hold or are you currently applying for any patents relating to the content of the manuscript?
- Have you received reimbursements, fees, funding, or salary from an organization that holds or has applied for patents relating to the content of the manuscript?
- Do you have any other financial competing interests?
- Do you have any non-financial competing interests in relation to this paper?

If you can answer no to all of the above, write 'I declare that I have no competing interests' below. If your reply is yes to any, please give details below.

I declare that I have no competing interests

I agree to the open peer review policy of the journal. I understand that my name will be included on my report to the authors and, if the manuscript is accepted for publication, my named report including any attachments I upload will be posted on the website along with the authors' responses. I agree for my report to be made available under an Open Access Creative Commons CC-BY license (http://creativecommons.org/licenses/by/4.0/). I understand that any comments which I do not wish to be included in my named report can be included as confidential comments to the editors, which will not be published.

Choose an item.

To further support our reviewers, we have joined with Publons, where you can gain additional credit to further highlight your hard work (see: https://publons.com/journal/530/gigascience). On publication of this paper, your review will be automatically added to Publons, you can then choose whether or not to claim your Publons credit. I understand this statement.

Yes Choose an item.
